# Supplementary material for: Belimumab after B cell depletion therapy in patients with systemic lupus erythematosus (BEAT Lupus) protocol: a prospective multicentre, double-blind, randomised, placebo-controlled, 52-week phase II clinical trial
Source: BMJ Open. 2019 Dec 16;9(12):e032569. doi: 10.1136/bmjopen-2019-032569 (PMC6937022; doi:10.1136/bmjopen-2019-032569)
Supplement: Supplementary data [file bmjopen-2019-032569supp001.pdf]

[to be printed on hospital headed paper]

Patient Information Sheet and informed consent form

Study title: BEAT LUPUS

Safety and efficacy of BElimumab After B cell depleTion therapy in systemic LUPUS erythematosus

Protocol Reference Number: CTU/2013/096

We would like to invite you to take part in our clinical study. University College London (UCL) is responsible for the study. Taking on this responsibility is called being the study sponsor. Before you decide whether or not to take part, we would like you to understand why the study is being done and what it would involve for you. One of our team will go through the information sheet with you and answer any questions you have. We'd suggest this should take about 20 minutes.
Ask us if there is anything that is not clear. Talk to others about the study if you wish. Take time to decide whether or not you wish to take part.

1. What is the purpose of the study?
This study aims to find out whether a drug called Belimumab (Benlysta) when used after B cell depletion therapy is both safe and effective in reducing systemic lupus erythematosus (lupus) disease activity. B cell depletion therapy (normally rituximab) is being given as part of your normal treatment because your lupus is active. B cell depletion therapy removes a type of immune cell called B cells from the body. B cells can cause disease in lupus patients. Although patients respond to B cell depletion therapy the disease can quickly return. This is likely to be because a chemical (also known as a stimulating factor) called BAFF increases in the body once treatment ends, and switches the lupus back on. Belimumab is a drug that stops BAFF from working and has been shown to work in patients with lupus. We will treat you with belimumab soon after B cell depletion therapy and we hope this combined treatment approach will prevent the lupus from coming back after rituximab therapy. However, we don't know yet whether giving belimumab is any better than simply having B cell depletion therapy alone.

2. Why have I been invited?
You have been invited because you have lupus and your disease has not responded to treatment with medication such as steroids and mycophenolate. Patients whose disease does not normally respond to these treatments can receive rituximab as part of their normal care. We aim to recruit 50 lupus patients to this study who have received B cell depletion therapy in the last month or so.

3. Do I have to take part?
It is up to you to decide to join the study. We will describe the study and go through this information sheet with you. If you agree to take part, we will then ask you to sign a consent form. You are free to withdraw your consent at any time without giving a reason. This will not affect the standard of care you receive.

Jones A, *et al. BMJ Open* 2019; 9:e032569. doi: 10.1136/bmjopen-2019-032569

4. What will happen to me if I take part?

If you wish to take part, you will be asked a series of questions to see if you might be eligible to join the study. If you are eligible at this point you will be asked to attend the hospital to be asked more details about your condition and be examined and have blood and urine tests.

If this assessment confirms you are eligible, you will be entered into the study and will be invited to attend the hospital again.

You will return to hospital to have further blood tests, and an ECG (a test of your heart). If you are a woman and of child bearing age then you will have a pregnancy test at each visit and as noted below you will be asked to use appropriate contraception so that you will not get pregnant whilst you are in the study and within 16 weeks after the last dose of study treatment. Tests will be run for serious infections such as HIV or Hepatitis. All these tests are tests you would have as part of your normal care.

This is a randomized study. We don‘t know the best way of treating patients after B cell depletion therapy. To find out, we need to compare a new treatment (belimumab) with usual treatment. We will put people into groups and give one group belimumab and the other group the usual treatment. The results are compared to see if one is better. To try to make sure the groups are the same to start with, each patient is put into a group by chance (randomly). The decision as to which group you will be put in will be made by a computer.

Half the patients receive belimumab and half receive placebo. Both treatments will be given by intravenous infusion. The placebo treatment will look the same as belimumab but contains no active drug. The reason for including a placebo is so neither you nor your doctor knows whether belimumab has been given. Sometimes simply knowing what treatment has been given can affect the way a patient is feeling. We will be able to compare the information from patients in the belimumab group, with the information from the placebo group and be able to determine what effect belimumab is having on Lupus.

Your doctor will be able to find out which group you are in should there be a need to, but this does not usually happen while you are taking part in the study.

All patients will be invited to attend for an infusion of the study medication which will last an hour (but you may be monitored for a few hours after the first couple of infusions) and you will repeat treatments and assessments, usually every four weeks.

The results of the study will be available for general use by researchers in the field of Lupus, to ensure that the information gathered from the study is put to maximum use. Your personal details will not be revealed at any stage.

5. Expenses and payments

We will pay your travel expenses as a result of taking part in the study.

6. What will I have to do?

If you join this study, you will need to come to the hospital 17 times over a period of 13 months as well as the visit when you decide whether you want to join the study.

We will ask you not to participate in studies of other therapies during the course of this study. There are no other restrictions on your lifestyle by being part of this study.

Jones A, *et al. BMJ Open* 2019; 9:e032569. doi: 10.1136/bmjopen-2019-032569

Your doctor can give you other treatments during the study such as prednisolone and other immunosuppressants with your agreement. Some of these treatments may be slowly reduced. You may need to remain on small doses of prednisolone and immunosuppressants until the end of the study. If your disease worsens during the study your doctor can increase these standard treatments with your agreement.

Trial Summary Assessments

Screening:

- Study discussion and patient consent
- Physical examination, review of medication, and blood tests
- Questionnaire completion
- Urine check
- Pregnancy check
- Research blood tests. These samples will be stored using a code and maybe sent to research labs outside of the hospital. All data are kept confidential.

Visit 1

- Physical examination, review of medication, and blood tests
- Questionnaire completion
- Urine check
- Pregnancy check
- ECG

Visit 2

- Physical examination, review of medication, and blood tests
- Questionnaire completion
- Urine check
- Pregnancy check
- Research blood tests. These samples will be stored using a code and maybe sent to research labs outside of the hospital. All data are kept confidential
- **Randomisation to Belimumab or placebo infusions**

Visits 3-16

Are at Week 2, 4, 8, 12, 16, 20, 24, 28, 32, 36, 40, 44, 48 and 52 weeks after randomisation

- Physical examination, review of medication, and blood tests
- Questionnaire completion
- Urine check
- Pregnancy check
- ECG (Visit 10,16)
- Research blood tests. These samples will be stored using a code and maybe sent to research labs outside the hospital. All data are kept confidential. The amount of blood taken will vary at each visit but will not be more than 120ml (about 6 tablespoons).
- Belimumab or placebo depending on which group you are in together with your usual treatments.

Follow up, Visit 17

Week 56

- Physical examination, review of medication, and blood tests
- Questionnaire completion
- Urine check
- Pregnancy check
- Research blood tests. These samples will be stored using a code and maybe sent to research labs outside the hospital. All data are kept confidential.

Jones A, *et al*. *BMJ Open* 2019; 9:e032569. doi: 10.1136/bmjopen-2019-032569

Visits 18
Week 68

- Pregnancy check
- Follow up phone call (male participants only)

Extra or unplanned visits
You may be asked to come for extra visits if your doctor believes your disease may be getting worse. If this happens you may be asked to have some of the tests that are usually performed at a study visit.

7. What are the alternatives for diagnosis or treatment?
The rituximab treatment can be repeated without belimumab. Prednisolone and other immunosuppressants can be used to treat your lupus.

8. What are the possible benefits of taking part?
During the study you will have a regular review of your Lupus by an experienced Lupus specialist. Two large studies each involving just over 800 patients with lupus have demonstrated that belimumab is an effective treatment for lupus resulting in fewer flares in disease and allows patients to reduce their steroid dose. It has not been studied in patients with severe lupus kidney disease or severe lupus disease affecting the brain. Other smaller studies suggest that belimumab after rituximab may be helpful to relieve some lupus symptoms and prevent lupus flares. We cannot promise the study will help you but the information we get from this study will help improve the treatment of lupus.

9. What are the possible disadvantages and risks of taking part?

The main risk of taking part is that belimumab has not been given after drugs like rituximab in a research study like this one before and we don't know if this is a safe combination.

Another disadvantage of taking part in this study is the need to spend approximately an hour reclining whilst having an infusion in to a vein in your arm on 15 occasions. If you feel that this would cause you too much discomfort, then you should not take part.

You will need to have blood tests at each assessment which carries the risk of bruising, discomfort or fainting.

10. What are the side effects of belimumab given soon after rituximab?
You should have been given appropriate information about your current treatment and if this has not happened please contact your local research team, they will be able to discuss details of potential side effects that might be associated with the treatment administered in BEAT Lupus.

Belimumab has been studied in patients with lupus but not after treatment with rituximab. Details of the possible side effects given below are taken from the results of two large trials of belimumab in lupus mentioned in part 8 of this leaflet. In those two trials belimumab caused few side effects and the number of infections was not increased compared to patients who received placebo. Patients given both rituximab followed by belimumab may experience worse or fewer side effects than either drug alone and some effects we may not be able to predict.

Serious Side Effects: Some patients receiving belimumab have experienced serious side effects. These have included:

Jones A, *et al. BMJ Open* 2019; 9:e032569. doi: 10.1136/bmjopen-2019-032569

Risk of Serious Allergic reactions:

The most common side effects that happen during or soon after a belimumab dose are nausea, diarrhoea, and fever. These side effects are usually not severe.

Some patients (up to 1 out of 100) have more severe side effects. These may happen because of an allergic reaction. Patients who have had allergic reactions before may be more likely to have allergic reactions to belimumab. These reactions happen most often on the day of or the day after the first or second belimumab dose. They can be very serious but rarely cause death. With these reactions there may be swelling of the face, lips, mouth, or tongue; wheezing, difficulty in breathing or shortness of breath; rash or itchy raised bumps (welts or hives), slow heartbeat, high or low blood pressure, or dizziness. Get medical help right away if you get any of these problems during the study.

Your study doctor or study nurse will watch you closely during and after each infusion for signs of a reaction. You will need to stay in the clinic for 3 hours after the first and second infusions. Your doctor may decide to watch you for more than 3 hours after a treatment or after more than 2 treatments. Sometimes allergy medicines or medicines like paracetamol are given before an infusion. It is not known if this stops you from having allergic side effects, or if it will make allergic side effects less severe.

Sometimes allergic side effects can occur after you have left the hospital. These types of reactions generally occur 5-10 days after a dose of medication (but can occur before or after that time) and may include a combination of symptoms such as rash, nausea, fatigue, muscle aches, headache, and/or facial swelling. If you experience these symptoms, particularly if you experience a combination of such symptoms, contact your study doctor or study nurse.

Risk of Infection

Because of the type of medicine it is, belimumab may make it more likely for you to get an infection. Tell your doctor if you get chills or a fever or any other sign that makes you think you may have an infection. If you have an infection that keeps coming back or is hard to get rid of, you should not be treated with belimumab. Make sure you talk to your study doctor if you have or get these kinds of infections.

Progressive multifocal leukoencephalopathy (PML) is a serious and life threatening brain condition. Your chance of getting PML may be higher if you are treated with medicines that weaken your immune system, including belimumab. Tell your doctor immediately if you have memory loss, trouble thinking, difficulty with talking or walking, loss of vision, or similar problems

Risk of Cancer

So far, in the many thousands of patients who have received belimumab the risk of cancer has not increased compared to patients who do not get belimumab. However, we cannot say for definite that belimumab does not increase the risk of cancer to a small degree.

Vaccines

Vaccines help your body fight infections. If you have a vaccine while you are taking belimumab, the vaccine might not work as well as if you weren't taking belimumab. Also, some types of vaccines may not be safe for you to have while you are taking belimumab. Make sure you check with your study doctor before having any vaccine.

Jones A, *et al. BMJ Open* 2019; 9:e032569. doi: 10.1136/bmjopen-2019-032569

Male fertility

Your doctor will explain that the effect on human male fertility is unknown, therefore if you are a male patient entering this trial, you must use a form of effective contraception throughout the trial and for at least 4 months after the last dose of study medicine. Your study doctor or nurse will phone you 16 weeks after your last trial treatment to obtain information as to whether there has been any reported pregnancies.

Pregnancy and Contraception

You should not take part in this study if you are pregnant. Mothers should not breastfeed a baby while on this study.

You should not get pregnant while you are in this study. If you are a woman who is able to get pregnant you must agree to use a type of birth control that works well. Your doctor will explain to you that the effects of the study treatment on pregnancy and the developing foetus are not yet known or are not fully understood at this time, therefore you will have to use one of these types of birth control while you are in this study and for at least 4 months after the last dose of study medicine. Your study doctor or study nurse will tell you about the choices of birth control that you can use in this study. Even if you are using birth control, you will have pregnancy testing at every visit during the study and up to 4 months after your last infusion of the study drug. Tell your study doctor or study nurse if you become pregnant during the study or within 4 months after the last infusion of the study drug...

If you have lupus and are taking a drug for your lupus called mycophenolate (for example CellCept®), additional methods of birth control will be required unless you avoid sexual intercourse completely (abstinence as defined above). Discuss required methods of contraception with your study doctor. Birth control pills may not work as well when you are taking mycophenolate and you could become pregnant.

Serious Mental Health Problems and Suicide:

Mental health problems, such as depression, suicide, trouble sleeping and anxiety are common in patients with lupus and were reported in patients receiving belimumab. Depression, thoughts of suicide and attempting to commit suicide have been reported more frequently in patients receiving belimumab than in patients who did not receive belimumab. Committing suicide has also been reported in patients who took belimumab.

Tell your study doctor or study nurse right away or go to a hospital right away if you attempt to commit suicide, action dangerous impulses, or have thoughts of suicide, hurting yourself, or dying. Also tell your doctor if you have been depressed, worried or anxious.. Tell your doctor even if these problems are not bothering you a lot right now. Also tell your study doctor if you are having any mood problems, are not acting or feeling like yourself, or are having behaviour problems. If your family or friends have told you that they think you have these problems, tell your doctor, even if you don't agree.

You will be asked questions about your mood and any thoughts of harming yourself that you may have had prior to or during the study.

You will be asked to complete a test administered by your study doctor or study nurse to evaluate your risk for suicide. Mental health problems such as depression, suicide, trouble sleeping, and anxiety are common in patients with lupus and were reported in patients receiving belimumab. Depression, thoughts of suicide and attempting to commit suicide have been reported more frequently in patients receiving belimumab than in patients who did not receive belimumab.

Jones A, *et al. BMJ Open* 2019; 9:e032569. doi: 10.1136/bmjopen-2019-032569

Committing suicide has also been reported in patients who took belimumab. You will be asked questions about your mood and any thoughts of self-harm that you may have. Tell your study doctor if you have new or worse depression or anxiety, thoughts about suicide or dying or other unusual changes in behaviour or mood during the study.

11. Combination of belimumab and rituximab

There is no available information from previous or ongoing clinical studies regarding the side effects of administering belimumab and rituximab in combination. Therefore, there is the possibility that the combination of these two medicines may increase the side effects potentially caused by either medicine alone or may cause new side effects that are not known now.

Both belimumab and rituximab may increase your risk of infection and/or suffering allergic side effects when the drug is given to you. It is possible that this risk may be increased when belimumab and rituximab are administered close together. Your doctor can explain to you the steps that will be taken to reduce this risk.

In addition, it is also possible that the risks of cancer and effects on your mood (feeling anxious or depressed) could be increased when both belimumab and rituximab are administered close together.

12. What happens when the research study stops?

Belimumab treatment will not be automatically provided to any patient participating in this study after its completion. All patients will continue to receive their regular lupus medication and follow up appointments with their referring doctor. Please note that patients will also remain under the study team for 30 days after stopping belimumab or placebo. It is also possible that you may receive more rituximab after the study as part of normal care or belimumab if these are available under the NHS. As belimumab is relatively expensive its use is currently restricted by the NHS, and it is not possible to predict whether an individual patient would be eligible to receive belimumab. The results of this study may help to make belimumab available after rituximab but only if the results show that belimumab after rituximab helps patients with lupus. As this is a small trial further studies may be needed.

It is possible that we may stop the study early because the results look like the belimumab is not working, or is unsafe. If this happens the reasons will be discussed with you and your doctor will talk to you about your treatment options.

13. What if there is a problem?

Every care will be taken in the course of this clinical study. However, in the unlikely event that you are injured by taking part, compensation may be available.

If you suspect that the injury is result of UCL's or your hospital's negligence then you may be able to claim compensation. After discussing with your clinical study doctor, you should make the claim in writing to your study doctor who will then pass the claim to the Sponsor's Insurers, via the Sponsor's office. You may have to bear the costs of the action initially, and you should consult a lawyer about this. Participants may also be able to claim compensation for injury caused by participation in this clinical study without the need to prove negligence on the part of University College London or another party. All claims should be made in writing to Professor Ehrenstein.

Regardless of this, if you wish to complain, or have any concerns about any aspect of the way you have been approached or treated by members of staff or about any side effects (adverse events) you may have experienced due to your participation in the study, the normal National Health Service complaints mechanisms are available to you. Please ask your study doctor if you would like more

information about this. Details can also be obtained from the Department of Health website: http://www.dh.gov.uk. Alternatively local contact details for complaints:

Name *[insert name]* **Tel. Number: *[insert number]***

14. What will happen to information about me collected during the trial?

University College London is the sponsor for this study based in the United Kingdom. We will be using information from you and/or your medical records in order to undertake this study and will act as the data controller for this study. This means that we are responsible for looking after your information and using it properly. University College London will keep identifiable information about you for a minimum of 10 years after the study has finished.

Your rights to access, change or move your information are limited, as we need to manage your information in specific ways in order for the research to be reliable and accurate. If you withdraw from the study, we will keep the information about you that we have already obtained. To safeguard your rights, we will use the minimum personally-identifiable information possible.

You can find out more about how we use your information at www.ucl.ac.uk/cctu/use-of-data

[insert name of NHS site] will collect information from you and/or your medical records for this research study in accordance with our instructions.

[insert name of NHS site] will keep your name, and contact details confidential and will not pass this information to University College London. ***[insert name of NHS site]*** will use this information as needed, to contact you about the research study, and make sure that relevant information about the study is recorded for your care, and to oversee the quality of the study. Certain individuals from University College London and regulatory organisations may look at your medical and research records to check the accuracy of the research study. University College London will only receive information without any identifying information. The people who analyse the information will not be able to identify you and will not be able to find out your name, or contact details.

[insert name of NHS site] will keep identifiable information about you from this study for 10 years after the study has finished.

When you agree to take part in a research study, the information about your health and care may be provided to researchers running other research studies in this organisation and in other organisations. These organisations may be universities, NHS organisations or companies involved in health and care research in this country or abroad. Your information will only be used by organisations and researchers to conduct research in accordance with the UK Policy Framework for Health and Social Care Research.

This information will not identify you and will not be combined with other information in a way that could identify you. The information will only be used for the purpose of health and care research, and cannot be used to contact you or to affect your care. It will not be used to make decisions about future services available to you, such as insurance.

15. What if relevant new information becomes available?

Sometimes we get new information about the treatment being studied. If this happens, we will tell you about it and discuss whether you want to or should continue in the study. If you decide not to carry on, we will make arrangements for your care to continue. If you decide to continue in the study we will ask you to sign an updated consent form.

Jones A, *et al. BMJ Open* 2019; 9:e032569. doi: 10.1136/bmjopen-2019-032569

16. What will happen if I don't want to carry on with the study?

You may withdraw from the study at any time without giving a reason. However, if a patient withdraws from a clinical study it can affect how the results are analysed, so we will ask you to think carefully about participating in the study and attending for all visits before you agree to take part. Information collected during the study may still be used. We will ask to continue to see you to monitor your progress but this is not compulsory. If you do not wish to continue follow up, we will request to see you for an exit check-up. Any stored blood samples that can still be identified as yours will be destroyed if you wish.

17. Will my GP be informed of my involvement?

With your consent, your GP, and other doctors who may be treating you, will be notified that you are taking part in this study. If there are any changes in your health requirements as a result of taking part in the study, your GP will again be informed. You can only join this study if you agree to your GP being informed.

18. What will happen to any samples I give?

Blood samples and urine samples will be collected at every study visit. Some of these samples will be tested at other research laboratories and then securely stored in case any need arises to test them as part of future research. These samples will be accessible only to the staff of the hospital laboratories. Those samples that are sent to research laboratories will be coded and there will be no patient identifiable information on these tubes. They will not be transferred out of the UK.

19. Will any genetic tests be done?

The tests on the research bloods may include genetic testing.

20. What will happen to the results of the research study?

The results of the study will be available after it finishes and will usually be published in a medical journal or presented at a scientific conference. The data will be anonymous and none of the patients involved in the study will be identified in any report or publication. Should you wish to see the results, or the publication, please ask your study doctor.

21. Who is organising and funding the research?

This research is being funded by Arthritis Research UK, GSK and UCLH Biomedical Research Centre, and the drug is being provided free of charge by GSK. The study is being co-ordinated by University College London Comprehensive Clinical Trials Unit. The doctors looking after you are not being paid for including you and have no conflicts of interest.

22. Who has reviewed the study?

All research in the NHS is looked at by an independent group of people, called a Research Ethics Committee, to protect your interests. This study has been reviewed and given favourable opinion by London - Hampstead Research Ethics Committee.

23. Further information and contact details

You are encouraged to ask any questions you wish, before, during or after your treatment. If you have any questions about the study, please speak to your study nurse or doctor, who will be able to provide you with up to date information about the drug involved. If you wish to read the research on which this study is based, please ask your study nurse or doctor. If you require any further information or have any concerns while taking part in the study please contact one of the following people:

Your Study Doctor

Name*:* ***[insert name]*** Tel. Number: ***[insert number]***

Jones A, *et al*. *BMJ Open* 2019; 9:e032569. doi: 10.1136/bmjopen-2019-032569

Your Research Nurse

Name; ***[insert name]*** Tel. Number: ***[insert number]***

Emergency Contact Number: [*insert number*]

If you decide you would like to take part then please read and sign the consent form. You will be given a copy of this information sheet and the consent form to keep. A copy of the consent form will be filed in your patient notes and one will be filed with the study records. You can have more time to think this over if you are at all unsure. Thank you for taking the time to read this information sheet and to consider this study.

Jones A, *et al. BMJ Open* 2019; 9:e032569. doi: 10.1136/bmjopen-2019-032569

[to be printed on hospital headed paper]

Study title: BEAT LUPUS- Safety and efficacy of BElimumab After B cell depleTion therapy in systemic LUPUS erythematosus

Protocol Reference Number: CTU/2013/096

Participant Identification Number: ___________

CONSENT FORM

Please initial each line

1	I have read the information sheet version ______ dated ________________for this study. I have had the opportunity to consider the information and ask questions that have been answered satisfactorily.	____________
2	I understand that my participation in this study is voluntary and that I am free to withdraw at any time, without giving any reason and without my medical care or legal rights being affected.	____________
3	I agree to my General Practitioner being informed of my participation in this study.	____________
4	I understand that relevant sections of my medical notes and data collected during the study may be looked at by individuals from the sponsor of the study (University College London), regulatory authorities, or from the NHS Trust, where it is relevant to my taking part in this research.	____________
5	I agree to take part in the above study.	____________
6	I agree that specimens collected as part of the above study may be transferred outside of the hospital in which they were taken, for storage and analysis for this and other ethically approved studies in the future.	____________
7	I agree to provide contact details for a family member, carer or friend to be used in case the study team are unable to contact me for follow up appointments.	____________

Name of Participant	Date	Signature
Name of Witness (where necessary)	Date	Signature
Name of Researcher	Date	Signature

When completed: 1 for participant; 1 for researcher site file (original); 1 to be kept in medical notes

Jones A, *et al. BMJ Open* 2019; 9:e032569. doi: 10.1136/bmjopen-2019-032569
