# Supplementary material for: Belimumab after B cell depletion therapy in patients with systemic lupus erythematosus (BEAT Lupus) protocol: a prospective multicentre, double-blind, randomised, placebo-controlled, 52-week phase II clinical trial
Source: BMJ Open. 2019 Dec 16;9(12):e032569. doi: 10.1136/bmjopen-2019-032569 (PMC6937022; doi:10.1136/bmjopen-2019-032569)
Supplement: Author's manuscript [file bmjopen-2019-032569.draft_revisions.pdf]

BMJ Open is committed to open peer review. As part of this commitment we make the peer review history of every article we publish publicly available.

When an article is published we post the peer reviewers' comments and the authors' responses online. We also post the versions of the paper that were used during peer review. These are the versions that the peer review comments apply to.

The versions of the paper that follow are the versions that were submitted during the peer review process. They are not the versions of record or the final published versions. They should not be cited or distributed as the published version of this manuscript.

BMJ Open is an open access journal and the full, final, typeset and author-corrected version of record of the manuscript is available on our site with no access controls, subscription charges or pay-per-view fees (http://bmjopen.bmj.com).

If you have any questions on BMJ Open's open peer review process please email info.bmjopen@bmj.com

BMJ Open

Belimumab after B cell depletion therapy in patients with systemic lupus erythematosus (BEAT LUPUS) trial protocol: A prospective multi-centred, double-blind, randomised, placebo-controlled, 52-week phase II clinical trial

Journal:	*BMJ Open*
Manuscript ID	bmjopen-2019-032569
Article Type:	Protocol
Date Submitted by the Author:	25-Jun-2019

Complete List of Authors:	Jones, Alexis; University College London, Division of Medicine<br>Muller, Patrick; University College London, Comprehensive Clinical Trials Unit<br>Dore, Caroline; MRC Clinical Trials Unit,<br>Ikeji, Felicia; University College London, Comprehensive Clinical Trials Unit<br>Caverly, Emilia; University College London, Comprehensive Clinical Trials Unit<br>Chowdhury, Kashfia; University College London, Comprehensive Clinical Trials Unit<br>Isenberg, David; Centre for Rheumatology Research, Division of Medicine UCL , Division of Medicine, UCL<br>Gordon, Caroline; Birmingham University Medical School, Rheumatology Research Group<br>Ehrenstein, MR; University College London, Division of Medicine

Keywords:	RHEUMATOLOGY, Rheumatology < INTERNAL MEDICINE, IMMUNOLOGY

SCHOLARONE™
Manuscripts

Belimumab after B cell depletion therapy in patients with systemic lupus erythematosus (BEAT LUPUS) trial protocol: A prospective multi-centred, double-blind, randomised, placebo-controlled, 52-week phase II clinical trial

Authors:

Alexis Jones[1], Patrick Muller[2], Caroline J Doré[2], Felicia Ikeji[2], Emilia Caverly[2], Kashfia Chowdhury[2], David A Isenberg[1], Caroline Gordon[3], and Michael R Ehrenstein[1].

[1] Department of Rheumatology, University College London Hospital, London, UK

[2] Comprehensive Clinical Trials Unit, University College London, London, UK

[3] Rheumatology Research Team - Inflammation and Ageing (IIA), University of Birmingham Research Laboratories, New Queen Elizabeth Hospital, Birmingham, UK

Correspondence to

Prof. Michael Ehrenstein

Department of Rheumatology

University College London

London

WC1E 6JF

Email: m.ehrenstein@ucl.ac.uk

Keywords: *systemic lupus erythematosus, B cell depletion, Rituximab, Belimumab, randomised clinical trial*

For peer review only

ABSTRACT

Introduction: Few treatment options exist for patients with systemic lupus erythematosus (SLE) who have failed conventional disease modifying drugs. Although widely used to treat moderate to severe lupus, the efficacy of B cell depletion therapy (BCDT) with Rituximab has not been demonstrated in randomised clinical trials. Following BCDT, elevated levels of serum B-cell activating factor (BAFF) have been associated with failure to remit or subsequent lupus relapse. The administration of Belimumab, a monoclonal antibody specific for BAFF and approved for lupus therapy, could potentiate the efficacy of Rituximab and enable longer periods of disease remission. The aim of this trial is to assess the safety and efficacy of Belimumab following Rituximab therapy in patients with SLE.

Methods and analysis: BEAT lupus is a double-blind, randomised, placebo controlled, phase II clinical trial. Patients receiving Rituximab as standard of care are randomised to receive Belimumab or placebo, 4 to 8 weeks following their first Rituximab infusion. Belimumab or placebo infusions are administered for 52 weeks. The primary outcome measure is anti-double stranded DNA (dsDNA) antibody levels at 52 weeks. Secondary outcomes include measures of adverse events, lupus disease activity and cumulative steroid dose. The kinetics of B cell repopulation and B and T cell phenotype and function during repopulation will be assessed in a subgroup of participants. Belimumab administration after Rituximab may provide a novel therapeutic pathway for patients with active lupus if safety is demonstrated in this proof of concept study, and lower anti-dsDNA antibodies levels are achieved in those patients on belimumab compared to placebo.

Ethics and Dissemination: Trial information is available at https://www.isrctn.com/ISRCTN47873003, and the results of this trial will be submitted for publication in relevant peer-reviewed publications. Key findings will also be presented at national and international conferences.

For peer review only

Strengths and limitations of this trial

- **This trial was developed as a direct result of experimental data suggesting that flares post-rituximab are driven by elevated BAFF levels. The experimental component of this trial will explore B and T cell kinetics, enabling a greater understanding of response or lack thereof to B cell depletion therapy.**

- **The trial design strictly limits steroid and immunosuppressant dosage, reflecting this unmet clinical need.**

- **It has a small sample size due to safety considerations prevailing at the conception of the trial.**

- **It was designed to test whether the UK can recruit sufficiently to a double blind randomised controlled trial in lupus.**

- **This trial looks at augmenting a treatment (Rituximab) which is already administered as part of standard of care according to NHS Commissioning policy.**

For peer review only

For peer review only - http://bmjopen.bmj.com/site/about/guidelines.xhtml

Introduction

Despite burgeoning research into the pathogenesis of systemic lupus erythematosus (SLE), the introduction of novel treatments has been slow. This is in contrast to some other immune mediated rheumatic diseases, in particular rheumatoid arthritis (RA), for which several novel therapies developed through increased understanding of disease pathogenesis have made a substantial impact upon patient care. A number of reasons may account for the disparity between SLE and RA including the greater use of corticosteroids which limits the potential for novel treatments to show superiority in clinical trials; the multisystem nature of lupus and insufficient understanding of the key factors that drive disease (1).

Rituximab, a chimeric anti-CD20 monoclonal antibody (mAb), has shown significant, though variable, efficacy in open-label studies, including both single-centre (2-5) and multicentre studies (6-8) (9); as well as a systematic review of off-label use (6). However, two large, phase III, randomised placebo-controlled trials in non-renal lupus (EXPLORER) (10, 11) and renal lupus (LUNAR) (12), failed to meet their primary end-points. Nevertheless, the European League Against Rheumatism (REF) have accepted that therapy with rituximab should be considered in organ-threatening, refractory lupus (13). In addition, NHS England have approved Rituximab as part of standard of care in patients with moderate to severe lupus who have failed conventional immunosuppressant therapy due to the few treatment options available for this patient group (NHS ENGLAND A13/PS/a)(14). However, the variable therapeutic success of rituximab highlights the need to refine therapies that target B cells to improve the outcome for patients with SLE.

The efficiency of B cell depletion following Rituximab treatment has been shown to predict response in patients with lupus (4, 15, 16). Analysis of peripheral B cell subsets revealed that patients with lower plasmablast counts at 6 months are more likely to have a sustained response without requiring retreatment (4). In the subset of SLE patients with high levels of anti-double stranded DNA (anti-dsDNA) antibodies, clinical relapse occurs at lower B cell numbers after treatment compared to before Rituximab and is associated with higher proportions of repopulating plasmablasts (15). Further investigation into the mechanism of Rituximab has demonstrated that levels of the B cell cytokine BAFF (B cell activating factor) surge after B cell depletion (17, 18). Moreover, elevated serum BAFF levels can endure beyond initial B cell repopulation and distinguish lupus relapse from ongoing disease remission following Rituximab (19). In some lupus patients with high levels of anti-dsDNA antibodies, repeated cycles of Rituximab resulted in ever higher levels of serum anti-dsDNA antibodies, which was associated with increasing levels of serum BAFF (16). This

For peer review only - http://bmjopen.bmj.com/site/about/guidelines.xhtml

effect raises the possibility that repeated Rituximab treatments may result in more severe flares driven by BAFF (20). Therefore, high BAFF levels post Rituximab could be limiting its effectiveness in some patients with SLE.

Clinical studies have demonstrated that BAFF is over-expressed, either intermittently or persistently, in many patients with SLE irrespective of Rituximab therapy (21, 22) and serum levels correlate with serologic and clinical indices of disease activity (23). In 2016, the BAFF-neutralizing monoclonal antibody, Belimumab was the first biologic licensed for the treatment of lupus following two large phase III clinical trials, BLISS 52 (24) and BLISS 76 (25). Treatment with Belimumab after B cell depletion has been suggested as a potential therapeutic strategy for patients with SLE (20). We therefore designed a phase II clinical trial to assess the safety and effectiveness of this treatment regime for patients with lupus, as judged by a change in anti-dsDNA antibody levels as the primary outcome. Anti-dsDNA antibodies have been associated with disease activity in patients with SLE (14, 26, 27). Secondary outcomes include reduction in cumulative steroid intake and incidence of disease flares during the 52-week follow-up period. This trial will also provide the opportunity to assess the kinetics of B cell repopulation, and B and T cell phenotype and function over the course of the trial.

For peer review only - http://bmjopen.bmj.com/site/about/guidelines.xhtml

Methods and analysis

BEAT lupus is a phase IIb, multicentre, UK based, randomised, double blind, placebo-controlled clinical trial investigating the safety and efficacy of Belimumab given for 12 months, with randomisation starting 4-8 weeks after a single course of B cell depletion therapy (Rituximab) given according to NHS England interim commissioning policy (Figure 1) (14). The target population of the trial is patients with a diagnosis of lupus undergoing Rituximab therapy as standard of care. The trial began recruitment in November 2016 and closed for recruitment in March 2019, with data collection of the primary outcome expected to end in March 2020. Collection of safety data will end in April 2020.

Study population

Detailed inclusion and exclusion criteria are listed in Box 1 In brief, patients are aged between 18 and 75 years with 4 or more criteria for SLE, according to the American College of Rheumatology (ACR) 1997 criteria or SLICC 2012 criteria for lupus, or biopsy proven lupus nephritis with one additional supportive test on at least two occasions (positive ANA, anti-dsDNA antibodies or anti-Sm antibodies) (14). Patients had to have a history of anti-dsDNA antibodies detectable at least once in the past 5 years. Participants had to be eligible for treatment with Rituximab as per NHS England Commissioning Policy. A screening and assessment visit are performed before the first Rituximab infusion, which is 4-8 weeks before randomisation (Day 0). A second screen is performed no more than 10 days before randomisation to confirm eligibility (e.g. excluding patients who required intravenous antibiotics for infections developing after rituximab or low IgG levels, see Box 1).

Randomisation and Blinding

BEAT Lupus is a double blind trial. Only the local pharmacist, the unblinded site monitor and the trial statistician will be unblinded to the treatment allocation. All other members of the trial team and patients are blinded to the trial medication throughout the trial. Unblinding will only occur if a participant experiences a serious adverse event for which the clinical management of the SAE will be facilitated by the unblinding of the participant's treatment allocation.

Randomisation was performed using minimisation, incorporating a random element to ensure balance in the stratifying variables between the two randomised groups, while retaining an element of unpredictability. Stratifying variables are: CD19 count after rituximab at randomisation ($<0.01\times10^9$/l vs $>0.01\times10^9$/l) to account for variability in B cell depletion which would affect

For peer review only

response; anti dsDNA (positive or negative at first screen); and whether patients have active renal disease at screening.

Trial treatment

Participants either receive intravenous Belimumab or placebo 4-8 weeks after the first infusion of B cell depletion therapy (Rituximab). The total treatment period (Belimumab or placebo) will be 52 weeks (last infusion of belimumab or placebo at week 52) followed by a safety visit at week 56, and pregnancy test at week 68. In the active therapy arm, participants will receive Belimumab according to the standard dosage regime. The recommended dosage regimen is 10 mg/kg at 2-week intervals for the first 3 doses and at 4-week intervals thereafter. The participants in the placebo group receive the same volume of normal saline infusions (indistinguishable from active treatment) at the same time points as the active treatment group. Participants and physicians are blinded with respect to active treatment or placebo. The assessment schedule is summarised in Figure 1.

Concomitant medications

Before randomisation, which includes the period when Rituximab is administered, treatment is entirely at the physician's discretion, though all medication is recorded. From the day of randomisation (Day 0, see Figure 1), the maximum dose of Mycophenolate is 1g/day with a suggested dose reduction to 500mg/day at 3 months or as soon as possible thereafter. The maximum dose of azathioprine after randomization is 1mg/kg with a suggested dose reduction to 0.5mg/kg by 3 months or as soon as possible thereafter. The maximum dose of methotrexate allowed is 15mg/week with a dose reduction to 10mg/week by 3 months or as soon as possible thereafter. Only one of these three disease modifying drugs (Azathioprine, methotrexate and mycophenolate) can be administered post randomisation. No other immunosuppressants (e.g. cyclophosphamide, tacrolimus) are allowed after randomisation except in the case of severe flare. Background anti-malarial drugs are permitted but no new anti-malarials can be started after randomisation. Mepacrine is acceptable for skin problems resistant to Hydroxychloroquine and both can be combined if this regime was started before randomisation. No change in dose of the anti-malarials can be made after randomisation. Participants will be permitted to receive up to 20 mg prednisolone/day from randomisation. It is strongly encouraged that prednisolone should be reduced by 50% by 6 months post randomisation, with a maximum dose of 5mg/day recommended at 6 months if possible. Participants who flare with one BILAG A or two BILAG B scores (severe flare) will be permitted to receive other therapies or increased prednisolone at the discretion of the

For peer review only - http://bmjopen.bmj.com/site/about/guidelines.xhtml

treating physician and can also continue with Belimumab or placebo at the discretion of their physician.

Trial endpoints (Box 2)

The primary outcome measure is anti-dsDNA antibody levels at 52 weeks. Anti-dsDNA antibodies often correlate with disease activity (26, 27) (14). This biomarker was also chosen due its correlation with BAFF levels in participants post B cell depletion therapy (19). Serum anti-dsDNA antibodies levels will be verified in the laboratory at UCL.

Secondary outcome measurements include anti-dsDNA antibody levels at 24 weeks and the proportion of participants with serious adverse events. The proportion of participants with a severe disease flare (defined as 1 BILAG A or 2 BILAG B flares) by 24 and 52 weeks following randomisation will be assessed, as will the time to a severe disease flare. Participants who have a severe disease flare will be permitted to receive other therapies or increased prednisolone (including intravenous methylprednisolone/above baseline dose of Prednisolone/Mycophenolate/Azathioprine) at the discretion of the treating physician and can also continue with Belimumab or placebo at the discretion of their physician. Further cycles of Rituximab are not advised during the trial even in the event of a severe disease flare. Patients who have a severe disease flare will be invited to an additional visit where standard trial data, including serum for the primary endpoint analysis, is collected.

Disease activity will be assessed using the BILAG disease activity index and SLEDAI 2000. C3 and immunoglobulin levels will be recorded. As part of the assessment of response to treatment, we shall calculate the cumulative steroid dose during treatment from randomisation and the proportion of participants decreasing their baseline steroid dose by 50% without flaring from randomisation; or if below 10mg/day at baseline reducing steroid dose to 5mg/day or who discontinue glucocorticoids with stable disease. Lupus Quality of Life (Lupus QoL), SF-36 and EQ5D will be used as patient reported outcome measures. C-SSRS (Columbia suicide severity rating scale) will be used to assess suicidality risk. The endpoints will be assessed using the measures in Box 2.

Trial sample size calculation

The primary outcome is the estimated difference in anti-dsDNA binding antibody levels at 52 weeks, adjusted for anti-dsDNA binding levels at baseline. We aim to recruit up to 56 SLE participants (the original sample size of 50 was increased to account for a higher than expected dropout rate) and

For peer review only - http://bmjopen.bmj.com/site/about/guidelines.xhtml

randomise 28 participants to receive Belimumab and Rituximab and 28 participants to receive placebo and Rituximab. This will be sufficient to detect a difference between groups of 1.2 in log anti-dsDNA binding antibody levels (equivalent to a 232% increase in either group) at the 5% level of significance with 80% power. The sample size calculation was done using STATA 13. The calculation assumed anti-dsDNA binding antibody levels are log-normally distributed; a standard deviation in the outcome on the log scale of 1.7 (19); a correlation of 0.55 between baseline and 52-week measurements; and 20% loss to follow up by 52 weeks (increased from 10% due to a higher than expected dropout rate).

Statistical analysis

A CONSORT diagram will be used to describe the course of participants through the trial. Baseline characteristics will be summarised by treatment group.

The analysis of the primary outcome will be done using linear regression to estimate the difference in log anti-dsDNA levels between the two groups at 52 weeks, adjusting for the stratifying variables anti-dsDNA (at screening, pre-Rituximab), BILAG category (renal, non-renal) (at screening, pre-Rituximab), and CD19 count (at randomisation). The analysis will be done on the intention-to-treat principle; all available 52 week measurements will be included regardless of patient's adherence to their randomised treatment. A supportive sensitivity analysis will be done to estimate the treatment effect for only those patients who adhered to their allocated treatment for 52 weeks, and mediation analyses will be done to evaluate whether the observed effect of Belimumab on anti-dsDNA levels is mediated by differences between groups in the concomitant medication doses following randomisation.

A similar modelling approach will be adopted for the analysis of other continuous outcomes measured at baseline and at 52 weeks the trial. Fisher's exact test will be used to compare proportions. Two sample t tests or Mann Whitney U tests, depending on the distribution of the data, will be used to evaluate continuous outcomes compared only at the end of the trial. Kaplan-Meier survival curves will be produced for comparisons of time to an event.

Ethical considerations

This trial has been approved by the local ethics review board and the MHRA and will be conducted in accordance with ethical principles derived from Good Clinical Practice Guidelines, including the Declaration of Helsinki. Written informed consent will be collected from all patients by approved

For peer review only

study site nurse or physician. Patients will be assigned a participant identification number and all patient information transferred to the sponsor will contain this number, patient initials and date of birth. All Serious Adverse Events (SAE) will be reported to the sponsor within 24 hours. Protocol version 5 dated 21st January 2019 is the current protocol version and has been approved.

Dissemination

Trial information is available at https://www.isrctn.com/ISRCTN47873003 including study sites all of which are in England, and the results of this trial will be submitted for publication in relevant peer-reviewed publications. Key findings will also be presented at national and international conferences. Published results will be disseminated to investigators at participating sites, who will further disseminate the results to trial participants on request.

Patient and public involvement

Feedback gathered from focused patient involvement meetings was used to inform the design of this trial. Patients recognised the need for improving therapies for SLE. They suggested that the inclusion and exclusion criteria should not represent a significant barrier to recruitment and that the trial should be as close to “real-world” practise as possible. Two patient advisors are members of the Trial Steering Committee.

For peer review only - http://bmjopen.bmj.com/site/about/guidelines.xhtml

Discussion

This clinical trial has been developed through investigation and analysis of clinical and laboratory data of lupus patients receiving Rituximab. In particular, we noted increased serum BAFF levels correlated with a rise in anti-DNA antibodies at the time of flare in lupus patients post Rituximab (19). We therefore predicted that lupus patients treated with Belimumab following Rituximab therapy will have lower anti-dsDNA antibody levels compared to those that have received Rituximab alone, and that this combination would have greater efficacy than Rituximab alone. Given the paucity of novel therapies, and the many clinical trials that have failed to show efficacy for SLE, a combination of biologic therapies with complementary effects given in succession may be required to control disease. If the results of this trial are promising, a larger trial will be required to achieve sufficient power to detect improved clinical outcomes. Indeed, a larger trial is already underway (BLISS BELIEVE NCT03312907) testing whether rituximab given after belimumab confers an additional benefit compared to belimumab alone (28). The order of administration of these two biologics is reversed compared to BEAT-LUPUS and there is no Rituximab alone arm. The administration of Rituximab first allows for the detection of adverse events that occur in the 4-8 week period after Rituximab and before randomisation. A second screen 7-10 days before randomisation will exclude patients based on these adverse events. CALIBRATE (NCT02260934), a clinical trial for renal lupus uses a similar sequence of these two biologics to the trial reported here, though cyclophosphamide is administered with the rituximab, and higher doses of concomitant medications are allowed.

Recruitment to lupus trials has been challenging in the UK, and few academic led interventional SLE trials in the UK have met their recruitment target. The last academic led lupus trial in the UK not subject to early termination because of poor recruitment commenced in 1997 and completed recruitment in 2005 (though even this open label trial's recruitment target had to be reduced due to slow recruitment) (29). We endeavoured to optimise recruitment by designing a protocol that would be as close to real life clinical practice as possible. Consequently, there are no therapeutic restrictions before patients are randomised (to receive either Belimumab or placebo) giving therapeutic freedom to the treating physician including the possibility of administering high dose corticosteroids in conjunction with Rituximab. Rituximab had to be administered 4-8 weeks before randomisation and is administered in accordance with the NHS England's Interim Commissioning Policy (NHS ENGLAND A13/PS/a) for patients with refractory lupus (14). This policy only allows Rituximab to be given to patients who have failed to respond adequately to either mycophenolate or cyclophosphamide, unless contraindicated, or requiring unacceptably high doses of corticosteroids. Thus, the results of this trial will be relevant to lupus patients that are refractory to or intolerant of

For peer review only - http://bmjopen.bmj.com/site/about/guidelines.xhtml

conventional therapy, the patient group for which this combination therapy is most likely to be approved. The trial provides information about rates of recruitment to lupus trials in England which could inform the development and feasibility of other UK based trials planned in the future. Recruitment was aided through NHS England's policy applicable to biological therapies for SLE which encourages physicians and their patients to consider interventional clinical trials (NHS ENGLAND A13/PS/a) (14).

The trial protocol offers physicians some flexibility with the use of concomitant immunosuppressant and oral prednisolone after randomisation. However, because of safety concerns, and to maximise the chances of revealing any potential difference between the two treatment arms, corticosteroids are limited to a maximum dose of 20mg/day at randomisation and it is strongly encouraged to taper the dose by at least 50% by 6 months. Moreover, immunosuppressant doses are also restricted. It can be difficult to achieve reductions in both corticosteroids and immunosuppressants in patients who, by the nature of the inclusion criteria, are more resistant to therapy. Our strategy has been to advise that reduction in corticosteroid dose should be prioritised over tapering immunosuppressants due to the former's more problematic side effects, and its ability to mask the difference between treatment arms in lupus trials.

This trial has some limitations. Firstly, the sample size is relatively small because at the time of the trial's inception there were no published safety data on the combination of Rituximab and Belimumab and an earlier phase, smaller trial was advised. Therefore, the trial is powered on anti-DNA antibodies rather than a clinical endpoint. To limit the risk of adverse events due to immunosuppression, the dose of concomitant DMARDs is lower than routinely prescribed for patients with active disease, particularly renal patients receiving mycophenolate. This may have reduced recruitment of patients with active nephritis by some physicians, although this restriction in dosage could also be an advantage with respect to distinguishing between active drug and placebo. The serological inclusion criteria enable patients with a confirmed positive dsDNA antibody within the last 5 years to participate in the trial rather than patients who have raised anti-DNA antibodies at screen. This approach was taken to maximise recruitment but also with the recognition that flares post Rituximab can, in a proportion of patients, be associated with increased DNA antibody levels above that observed when Rituximab is first commenced (15).

The development of this trial has arisen directly from analysis of the immunologic changes following B cell depletion therapy. Building on this experimental medicine approach, analysis of B and T cell phenotype has been incorporated into this trial to understand how sequential targeting of B cells

For peer review only - http://bmjopen.bmj.com/site/about/guidelines.xhtml

and BAFF affect B cell numbers and phenotype. An exploratory analysis will aim to determine if elevated serum autoantibodies and BAFF, present at baseline or early during treatment, can predict an improved response to Belimumab after Rituximab. If the overall results from this trial are encouraging, a larger definitive trial using time to flare as the primary outcome may be considered after taking into account the results from other similar trials currently underway. The increasing interest in this combination therapy has the potential to offer a novel treatment option for patients with active lupus.

Funding

This trial is supported by Versus Arthritis (grant number 20873) and the UCLH Biomedical Research Centre (BRC). GSK are providing Belimumab free of charge, as well as additional funding. The MRC (MASTERPLANS CONSORTIUM) is supporting some of the experimental medicine applied to samples from this trial.

Conflict of interest statement

MRE has received grant/research support from GSK. CG has been a member of the speakers bureau for GSK and has received consultancy fees for attending advisory boards.

Contributorship

MRE conceived of the study, MRE, CG, CD and DAI initiated the study design. PM and KC and CD provided statistical expertise in clinical trial in clinical trial design. MRE, CG, CD, FI and EC assisted with protocol development. MRE, DAI, CG and CD are grant holders.

Acknowledgements

We acknowledge the important contribution of the BEAT Lupus Trial Steering Committee, NIHR, the Data Monitoring Committee, and all the patients participating in the trial or involved in its development. All authors reviewed and edited the draft version of the manuscript and approved the final version. All authors have approved the final manuscript submitted. We are indebted to the BEAT-LUPUS Trial Collaborators at the recruiting sites and British Isles Lupus Assessment Group (BILAG). The trial is sponsored by University College London, Gower Street London, WC1E 6BT, United Kingdom, +44 20 7679 6163, ctu.beatlupus@ucl.ac.uk.

Trial registration number: ISRCTN47873

https://www.isrctn.com/ISRCTN47873003

For peer review only - http://bmjopen.bmj.com/site/about/guidelines.xhtml

BOX 1 Patient inclusion and exclusion criteria
(randomisation to trial (belimumab or placebo occurs at Day 0)
Inclusion criteria

1. Age between 18 and 75 years
2. Participants with 4 or more criteria for SLE according to the American College of Rheumatology (ACR) 1997 criteria or SLICC 2012 criteria or biopsy proven lupus nephritis with one additional supportive test on at least two occasions (positive ANA, anti-dsDNA antibodies or anti-Sm antibodies).
3. History of anti-dsDNA antibodies detectable at least once in the past 5 years prior to screening the participant on the trial protocol (ELISA test is preferable for anti-dsDNA antibody testing).
4. Participants are due to be treated with the first infusion of B cell depletion therapy (Rituximab) 4-8 weeks before randomisation (Day 0). Previous use of Rituximab is allowed prior to this cycle.
5. No contraindications to the use of Belimumab.
6. Ability to provide informed consent

Exclusion criteria

1. Severe “critical” SLE flare defined as BILAG A flare in CNS system or any SLE manifestation requiring more immunosuppression than allowed within the protocol in the physician's opinion
2. Pregnancy and/or breastfeeding patients
3. At risk of pregnancy and unwilling to use an acceptable form of birth control contraception
4. Prior use of Belimumab, Atacicept or any biologic therapy (except Rituximab, but no other B cell depleting therapies)
5. Participation in any other interventional trial within the last 6 months
6. eGFR <30mls/min at screening
7. Active infections, including but not limited to:
   - Current or past infection with hepatitis B or C as defined by:
     Hepatitis B surface antigen positive
     Hepatitis B surface antibody positive and hepatitis B core antibody positive
     Hepatitis C antibody positive
   - Historically positive HIV test or test positive at screening for HIV
   - Active TB.
8. Infection history:
   - Currently on any suppressive therapy for a chronic infection (such as tuberculosis, pneumocystis, cytomegalovirus, herpes simplex virus, herpes zoster and atypical mycobacteria)
   - Hospitalization for treatment of infection within 60 days of Day 0
   - Use of parenteral (IV or IM) antibiotics (anti-bacterials, antivirals, anti-fungals, or anti-parasitic agents) within 30 days of Day 0
   - Receipt of a live-attenuated vaccine within 3 months of Day 0
   - In the investigator’s opinion, participants that are at high risk for infection (including but not limited to in dwelling catheter, dysphagia with aspiration, decubitus ulcer, history of prior aspiration pneumonia or recurrent severe urinary tract infection)
9. IgG levels below 4.0 g/L, IgA level < 10 mg/dL (IgG and IgA test must be performed no more than 10 days before Day 0
10. Primary immunodeficiency
11. History of malignant neoplasm within the last 5 years
12. History of cervical dysplasia CIN Grade III cervical high-risk human papillomavirus or abnormal cervical cytology other than abnormal squamous cells of undetermined significance (ASCUS) within the past 3 years. The participant will be eligible after the condition has resolved (e.g., follow-up HPV test is negative or cervical abnormality has been effectively treated >1 year ago)
12. Severe, progressive, or uncontrolled renal, hepatic, haematological, gastrointestinal, pulmonary, cardiac, or neurological disease or, in the investigator’s opinion, any other concomitant medical condition or significant abnormal laboratory value that places the participant at risk by participating in this trial with the exception of diseases or conditions related to active SLE.
13. Comorbidities not lupus related currently requiring systemic corticosteroid therapy.
14. Evidence of serious suicide risk including any history of suicidal behaviour in the last 6 months and/or any suicidal ideation in the last 2 months or who in the investigator’s judgement, pose a significant risk.
15. History of an anaphylactic reaction to parenteral administration of contrast agents, human or murine proteins or monoclonal antibodies.
16. Current drug or alcohol abuse or dependence, or a history of drug or alcohol abuse or dependence within 364 days prior to Day 0.
17. White blood cells (WBC) <1.5 x 10^9/L, Neutrophils <1 x 10^9/L measured up to 10 days before Day 0
18. A history of major organ transplant or haematopoietic stem/cell/marrow transplant or renal transplant.

BOX 2: OUTCOME MEASURES

Primary outcome measures
Anti-dsDNA-antibody levels at 52 weeks.
Secondary outcome measures

1. Anti-dsDNA antibody levels at 24 weeks
2. Proportion of participants with any adverse events by 52 weeks.
3. Proportion of participants with any serious adverse events by 52 weeks.
4. Proportion of participants with any infections by 52 weeks.
5. Proportion of participants with a severe disease flare (defined
as one BILAG A or two B flares) by 52 weeks.
6. Proportion of participants with a severe or a moderate disease flare (defined at least one BILAG B flare accompanied by an increase in concomitant lupus medication - glucocorticoids, Mycophenolate, Azathioprine, or Methotrexate) by 52 weeks.
7. Time to severe disease flare.
8. Proportion of participants with a severe disease flare by 24 weeks.
9. SLEDAI 2000 at 52 weeks.
10. Subject Global Assessment of Disease Activity (SGADA) at 52 weeks.
11. C3 levels at 52 weeks.
12. Immunoglobulin levels at 52 weeks.
13. Cumulative steroid dose from randomisation to 52 weeks.
14. Proportion of participants decreasing steroid dose at randomisation by 50% without experiencing a severe flare, or if below 10mg/day at randomisation reducing dose to 5mg/day or less at 52 weeks.
15. Lupus Quality of Life (Lupus QoL), SF-36 at 52 weeks
16. Average EQ-5D from randomisation to 52 weeks.
17. C-SSRS (Columbian Suicide Severity Rating Scale) to assess suicidality risk at 52 weeks.

Exploratory outcomes

1. BAFF (measurement of RNA from whole blood) at 52 weeks.
2. Kinetics of B cell repopulation, B and T cell phenotype and function during repopulation using

For peer review only - http://bmjopen.bmj.com/site/about/guidelines.xhtml

Figure 1 Schedule of Assessments

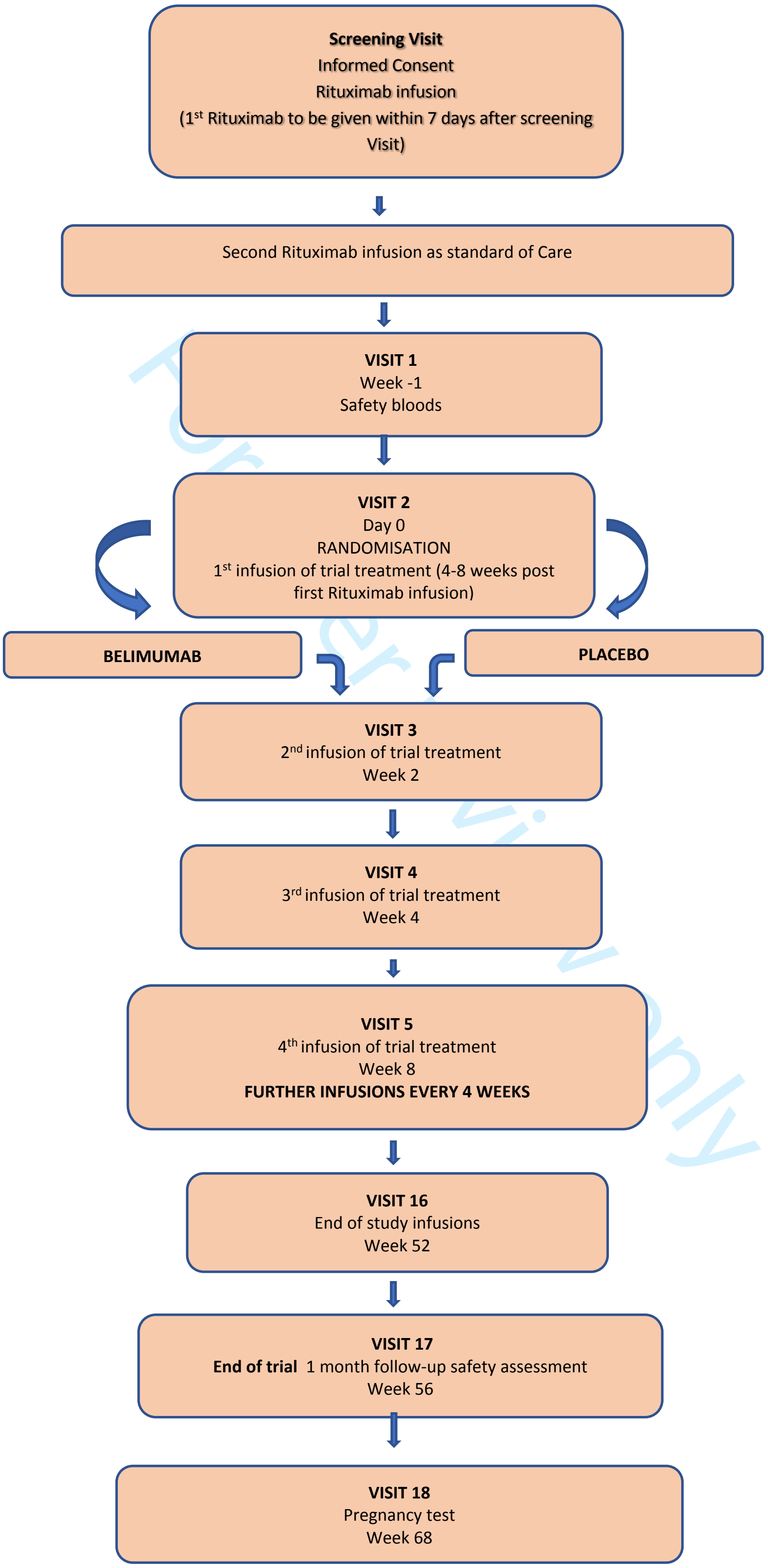

For peer review only

For peer review only - http://bmjopen.bmj.com/site/about/guidelines.xhtml

REFERENCES

1. Isenberg DA, Merrill JT. Why, why, why de-lupus (does so badly in clinical trials). Expert Rev Clin Immunol. 2016;12(2):95-8.
2. Galarza-Maldonado C, Kourilovitch MR, Molineros JE, Cardiel MH, Zurita L, Soroka NF, et al. The administration of low doses of rituximab followed by hydroxychloroquine, prednisone and low doses of mycophenolate mofetil is an effective therapy in Latin American patients with active systemic lupus erythematosus. Autoimmunity reviews. 2010;10(2):108-11.
3. Leandro MJ, Edwards JC, Cambridge G, Ehrenstein MR, Isenberg DA. An open study of B lymphocyte depletion in systemic lupus erythematosus. Arthritis & Rheumatism. 2002;46(10):2673-7.
4. Vital EM, Dass S, Buch MH, Henshaw K, Pease CT, Martin MF, et al. B cell biomarkers of rituximab responses in systemic lupus erythematosus. Arthritis & Rheumatism. 2011;63(10):3038-47.
5. Aguiar R, Araújo C, Martins-Coelho G, Isenberg D. Use of Rituximab in Systemic Lupus Erythematosus: A Single Center Experience Over 14 Years. Arthritis Care & Research. 2017;69(2):257-62.
6. Ramos-Casals M, Brito-Zerón P, Muñoz S, Soto M-J. A systematic review of the off-label use of biological therapies in systemic autoimmune diseases. Medicine. 2008;87(6):345-64.
7. Díaz-Lagares C, Croca S, Sangle S, Vital EM, Catapano F, Martínez-Berriotxoa A, et al. Efficacy of rituximab in 164 patients with biopsy-proven lupus nephritis: pooled data from European cohorts. Autoimmunity reviews. 2012;11(5):357-64.
8. Witt M, Grunke M, Proft F, Baeuerle M, Aringer M, Burmester G, et al. Clinical outcomes and safety of rituximab treatment for patients with systemic lupus erythematosus (SLE)–results from a nationwide cohort in Germany (GRAID). Lupus. 2013;22(11):1142-9.
9. McCarthy EM, Sutton E, Nesbit S, White J, Parker B, Jayne D, et al. Short-term efficacy and safety of rituximab therapy in refractory systemic lupus erythematosus: results from the British Isles Lupus Assessment Group Biologics Register. Rheumatology. 2017;57(3):470-9.
10. Merrill J, Buyon J, Furie R, Latinis K, Gordon C, Hsieh H, et al. Assessment of flares in lupus patients enrolled in a phase II/III study of rituximab (EXPLORER). Lupus. 2011;20(7):709-16.
11. Merrill JT, Neuwelt CM, Wallace DJ, Shanahan JC, Latinis KM, Oates JC, et al. Efficacy and safety of rituximab in moderately-to-severely active systemic lupus erythematosus: the randomized, double-blind, phase II/III systemic lupus erythematosus evaluation of rituximab trial. Arthritis & Rheumatism: Official Journal of the American College of Rheumatology. 2010;62(1):222-33.
12. Rovin BH, Furie R, Latinis K, Looney RJ, Fervenza FC, Sanchez-Guerrero J, et al. Efficacy and safety of rituximab in patients with active proliferative lupus nephritis: the Lupus Nephritis Assessment with Rituximab study. Arthritis & Rheumatism. 2012;64(4):1215-26.
13. Fanouriakis A, Kostopoulou M, Alunno A, Aringer M, Bajema I, Boletis JN, et al. 2019 update of the EULAR recommendations for the management of systemic lupus erythematosus. Annals of the Rheumatic Diseases. 2019;78(6):736-45.
14. Gordon C, Amissah-Arthur M-B, Gayed M, Brown S, Bruce IN, D'Cruz D, et al. The British Society for Rheumatology guideline for the management of systemic lupus erythematosus in adults. Rheumatology. 2017;57(1):e1-e45.

For peer review only

15. Lazarus MN, Turner-Stokes T, Chavele K-M, Isenberg DA, Ehrenstein MR. B-cell numbers and phenotype at clinical relapse following rituximab therapy differ in SLE patients according to anti-dsDNA antibody levels. Rheumatology. 2012;51(7):1208-15.
16. Iaccarino L, Bartoloni E, Carli L, Ceccarelli F, Conti F, De SV, et al. Efficacy and safety of off-label use of rituximab in refractory lupus: data from the Italian Multicentre Registry. Clinical and experimental rheumatology. 2015;33(4):449-56.
17. Cambridge G, Isenberg DA, Edwards J, Leandro MJ, Migone T-S, Teodorescu M, et al. B cell depletion therapy in systemic lupus erythaematosus: relationships among serum B lymphocyte stimulator levels, autoantibody profile and clinical response. Annals of the rheumatic diseases. 2008;67(7):1011-6.
18. Vallerskog T, Heimbürger M, Gunnarsson I, Zhou W, Wahren-Herlenius M, Trollmo C, et al. Differential effects on BAFF and APRIL levels in rituximab-treated patients with systemic lupus erythematosus and rheumatoid arthritis. Arthritis research & therapy. 2006;8(6):R167.
19. Carter LM, Isenberg DA, Ehrenstein MR. Elevated Serum BAFF Levels Are Associated With Rising Anti–Double-Stranded DNA Antibody Levels and Disease Flare Following B Cell Depletion Therapy in Systemic Lupus Erythematosus. Arthritis & Rheumatism. 2013;65(10):2672-9.
20. Ehrenstein MR, Wing C. The BAFFling effects of rituximab in lupus: danger ahead? Nature Reviews Rheumatology. 2016;12(6):367.
21. Cheema GS, Roschke V, Hilbert DM, Stohl W. Elevated serum B lymphocyte stimulator levels in patients with systemic immune–based rheumatic diseases. Arthritis & Rheumatism: Official Journal of the American College of Rheumatology. 2001;44(6):1313-9.
22. Stohl W, Metyas S, Tan SM, Cheema GS, Oamar B, Xu D, et al. B lymphocyte stimulator overexpression in patients with systemic lupus erythematosus: longitudinal observations. Arthritis & Rheumatism: Official Journal of the American College of Rheumatology. 2003;48(12):3475-86.
23. Petri M, Stohl W, Chatham W, McCune WJ, Chevrier M, Ryel J, et al. Association of plasma B lymphocyte stimulator levels and disease activity in systemic lupus erythematosus. Arthritis & Rheumatism: Official Journal of the American College of Rheumatology. 2008;58(8):2453-9.
24. Navarra SV, Guzmán RM, Gallacher AE, Hall S, Levy RA, Jimenez RE, et al. Efficacy and safety of belimumab in patients with active systemic lupus erythematosus: a randomised, placebo-controlled, phase 3 trial. The Lancet. 2011;377(9767):721-31.
25. Furie R, Petri M, Zamani O, Cervera R, Wallace DJ, Tegzová D, et al. A phase III, randomized, placebo-controlled study of belimumab, a monoclonal antibody that inhibits B lymphocyte stimulator, in patients with systemic lupus erythematosus. Arthritis & Rheumatism. 2011;63(12):3918-30.
26. Isenberg D, Manson J, Ehrenstein M, Rahman A. Fifty years of anti-ds DNA antibodies: are we approaching journey's end? 2007.
27. Floris A, Piga M, Cauli A, Mathieu A. Predictors of flares in Systemic Lupus Erythematosus: Preventive therapeutic intervention based on serial anti-dsDNA antibodies assessment. Analysis of a monocentric cohort and literature review. Autoimmunity Reviews. 2016;15(7):656-63.
28. Teng YKO, Bruce IN, Diamond B, Furie RA, van Vollenhoven RF, Gordon D, et al. Phase III, multicentre, randomised, double-blind, placebo-controlled, 104-week study of subcutaneous belimumab administered in combination with rituximab in adults with systemic lupus erythematosus (SLE): BLISS-BELIEVE study protocol. BMJ Open. 2019;9(3):e025687.

For peer review only

29. Griffiths B, Emery P, Ryan V, Isenberg D, Akil M, Thompson R, et al. The BILAG multi-centre open randomized controlled trial comparing ciclosporin vs azathioprine in patients with severe SLE. Rheumatology. 2010;49(4):723-32.

For peer review only

For peer review only - http://bmjopen.bmj.com/site/about/guidelines.xhtml

SPIRIT
STANDARD PROTOCOL ITEMS: RECOMMENDATIONS FOR INTERVENTIONAL TRIALS

SPIRIT 2013 Checklist: Recommended items to address in a clinical trial protocol and related documents*

Section/item	Item No	Description	Page Number on which item is reported
**Administrative information**			
Title	1	Descriptive title identifying the study design, population, interventions, and, if applicable, trial acronym	1
Trial registration	2a	Trial identifier and registry name. If not yet registered, name of intended registry	2
	2b	All items from the World Health Organization Trial Registration Data Set	
Protocol version	3	Date and version identifier	9
Funding	4	Sources and types of financial, material, and other support	12
Roles and responsibilitie s	5a	Names, affiliations, and roles of protocol contributors	12
	5b	Name and contact information for the trial sponsor	12
	5c	Role of study sponsor and funders, if any, in study design; collection, management, analysis, and interpretation of data; writing of the report; and the decision to submit the report for publication, including whether they will have ultimate authority over any of these activities	
	5d	Composition, roles, and responsibilities of the coordinating centre, steering committee, endpoint adjudication committee, data management team, and other individuals or groups overseeing the trial, if applicable (see Item 21a for data monitoring committee)	
**Introduction**			

For peer review only - http://bmjopen.bmj.com/site/about/guidelines.xhtml

Background and rationale	6a	Description of research question and justification for undertaking the trial, including summary of relevant studies (published and unpublished) examining benefits and harms for each intervention	4
	6b	Explanation for choice of comparators	4-5
Objectives	7	Specific objectives or hypotheses	2
Trial design	8	Description of trial design including type of trial (eg, parallel group, crossover, factorial, single group), allocation ratio, and framework (eg, superiority, equivalence, noninferiority, exploratory)	6-10
**Methods: Participants, interventions, and outcomes**			
Study setting	9	Description of study settings (eg, community clinic, academic hospital) and list of countries where data will be collected. Reference to where list of study sites can be obtained	10
Eligibility criteria	10	Inclusion and exclusion criteria for participants. If applicable, eligibility criteria for study centres and individuals who will perform the interventions (eg, surgeons, psychotherapists)	14
Interventions	11a	Interventions for each group with sufficient detail to allow replication, including how and when they will be administered	7
	11b	Criteria for discontinuing or modifying allocated interventions for a given trial participant (eg, drug dose change in response to harms, participant request, or improving/worsening disease)	7
	11c	Strategies to improve adherence to intervention protocols, and any procedures for monitoring adherence (eg, drug tablet return, laboratory tests)	
	11d	Relevant concomitant care and interventions that are permitted or prohibited during the trial	7
Outcomes	12	Primary, secondary, and other outcomes, including the specific measurement variable (eg, systolic blood pressure), analysis metric (eg, change from baseline, final value, time to event), method of aggregation (eg, median, proportion), and time point for each outcome. Explanation of the clinical relevance of chosen efficacy and harm outcomes is strongly recommended	15

For peer review only - http://bmjopen.bmj.com/site/about/guidelines.xhtml

Participant timeline	13	Time schedule of enrolment, interventions (including any run-ins and washouts), assessments, and visits for participants. A schematic diagram is highly recommended (see Figure)	16, Figure 1
Sample size	14	Estimated number of participants needed to achieve study objectives and how it was determined, including clinical and statistical assumptions supporting any sample size calculations	8
Recruitment	15	Strategies for achieving adequate participant enrolment to reach target sample size	
**Methods: Assignment of interventions (for controlled trials)**			
Allocation:			
Sequence generation	16a	Method of generating the allocation sequence (eg, computer-generated random numbers), and list of any factors for stratification. To reduce predictability of a random sequence, details of any planned restriction (eg, blocking) should be provided in a separate document that is unavailable to those who enrol participants or assign interventions	6
Allocation concealment mechanism	16b	Mechanism of implementing the allocation sequence (eg, central telephone; sequentially numbered, opaque, sealed envelopes), describing any steps to conceal the sequence until interventions are assigned	
Implementation	16c	Who will generate the allocation sequence, who will enrol participants, and who will assign participants to interventions	
Blinding (masking)	17a	Who will be blinded after assignment to interventions (eg, trial participants, care providers, outcome assessors, data analysts), and how	6
	17b	If blinded, circumstances under which unblinding is permissible, and procedure for revealing a participant's allocated intervention during the trial	6
**Methods: Data collection, management, and analysis**			

Data collection methods	18a	Plans for assessment and collection of outcome, baseline, and other trial data, including any related processes to promote data quality (eg, duplicate measurements, training of assessors) and a description of study instruments (eg, questionnaires, laboratory tests) along with their reliability and validity, if known. Reference to where data collection forms can be found, if not in the protocol	
	18b	Plans to promote participant retention and complete follow-up, including list of any outcome data to be collected for participants who discontinue or deviate from intervention protocols	
Data management	19	Plans for data entry, coding, security, and storage, including any related processes to promote data quality (eg, double data entry; range checks for data values). Reference to where details of data management procedures can be found, if not in the protocol	
Statistical methods	20a	Statistical methods for analysing primary and secondary outcomes. Reference to where other details of the statistical analysis plan can be found, if not in the protocol	9
	20b	Methods for any additional analyses (eg, subgroup and adjusted analyses)	
	20c	Definition of analysis population relating to protocol non-adherence (eg, as randomised analysis), and any statistical methods to handle missing data (eg, multiple imputation)	
**Methods: Monitoring**			
Data monitoring	21a	Composition of data monitoring committee (DMC); summary of its role and reporting structure; statement of whether it is independent from the sponsor and competing interests; and reference to where further details about its charter can be found, if not in the protocol. Alternatively, an explanation of why a DMC is not needed	
	21b	Description of any interim analyses and stopping guidelines, including who will have access to these interim results and make the final decision to terminate the trial	

For peer review only - http://bmjopen.bmj.com/site/about/guidelines.xhtml

Harms	22	Plans for collecting, assessing, reporting, and managing solicited and spontaneously reported adverse events and other unintended effects of trial interventions or trial conduct	
Auditing	23	Frequency and procedures for auditing trial conduct, if any, and whether the process will be independent from investigators and the sponsor	
**Ethics and dissemination**			
Research ethics approval	24	Plans for seeking research ethics committee/institutional review board (REC/IRB) approval	9
Protocol amendments	25	Plans for communicating important protocol modifications (eg, changes to eligibility criteria, outcomes, analyses) to relevant parties (eg, investigators, REC/IRBs, trial participants, trial registries, journals, regulators)	
Consent or assent	26a	Who will obtain informed consent or assent from potential trial participants or authorised surrogates, and how (see Item 32)	9
	26b	Additional consent provisions for collection and use of participant data and biological specimens in ancillary studies, if applicable	
Confidentiality	27	How personal information about potential and enrolled participants will be collected, shared, and maintained in order to protect confidentiality before, during, and after the trial	
Declaration of interests	28	Financial and other competing interests for principal investigators for the overall trial and each study site	
Access to data	29	Statement of who will have access to the final trial dataset, and disclosure of contractual agreements that limit such access for investigators	
Ancillary and post-trial care	30	Provisions, if any, for ancillary and post-trial care, and for compensation to those who suffer harm from trial participation	
Dissemination policy	31a	Plans for investigators and sponsor to communicate trial results to participants, healthcare professionals, the public, and other relevant groups (eg, via publication, reporting in results databases, or other data sharing arrangements), including any publication restrictions	10

For peer review only

	31b	Authorship eligibility guidelines and any intended use of professional writers	
	31c	Plans, if any, for granting public access to the full protocol, participant-level dataset, and statistical code	
**Appendices**			
Informed consent materials	32	Model consent form and other related documentation given to participants and authorised surrogates	
Biological specimens	33	Plans for collection, laboratory evaluation, and storage of biological specimens for genetic or molecular analysis in the current trial and for future use in ancillary studies, if applicable	

*It is strongly recommended that this checklist be read in conjunction with the SPIRIT 2013 Explanation & Elaboration for important clarification on the items. Amendments to the protocol should be tracked and dated. The SPIRIT checklist is copyrighted by the SPIRIT Group under the Creative Commons "Attribution-NonCommercial-NoDerivs 3.0 Unported" license.

For peer review only

BMJ Open

Belimumab after B cell depletion therapy in patients with systemic lupus erythematosus (BEAT LUPUS) trial protocol: A prospective multi-centred, double-blind, randomised, placebo-controlled, 52-week phase II clinical trial

Journal:	*BMJ Open*
Manuscript ID	bmjopen-2019-032569.R1
Article Type:	Protocol
Date Submitted by the Author:	09-Sep-2019

Complete List of Authors:	Jones, Alexis; University College London, Division of Medicine<br>Muller, Patrick; University College London, Comprehensive Clinical Trials Unit<br>Dore, Caroline; MRC Clinical Trials Unit,<br>Ikeji, Felicia; University College London, Comprehensive Clinical Trials Unit<br>Caverly, Emilia; University College London, Comprehensive Clinical Trials Unit<br>Chowdhury, Kashfia; University College London, Comprehensive Clinical Trials Unit<br>Isenberg, David; Centre for Rheumatology Research, Division of Medicine UCL , Division of Medicine, UCL<br>Gordon, Caroline; Birmingham University Medical School, Rheumatology Research Group<br>Ehrenstein, MR; University College London, Division of Medicine

**Primary Subject Heading**:	Rheumatology
Secondary Subject Heading:	Immunology (including allergy)
Keywords:	RHEUMATOLOGY, Rheumatology < INTERNAL MEDICINE, IMMUNOLOGY

SCHOLARONE™
Manuscripts

Belimumab after B cell depletion therapy in patients with systemic lupus erythematosus (BEAT LUPUS) trial protocol: A prospective multi-centred, double-blind, randomised, placebo-controlled, 52-week phase II clinical trial

Authors:

Alexis Jones[1], Patrick Muller[2], Caroline J Doré[2], Felicia Ikeji[2], Emilia Caverly[2], Kashfia Chowdhury[2], David A Isenberg[1], Caroline Gordon[3], and Michael R Ehrenstein[1].

[1] Department of Rheumatology, University College London Hospital, London, UK

[2] Comprehensive Clinical Trials Unit, University College London, London, UK

[3] Rheumatology Research Team - Inflammation and Ageing (IIA), University of Birmingham Research Laboratories, New Queen Elizabeth Hospital, Birmingham, UK

Correspondence to

Prof. Michael Ehrenstein

Department of Rheumatology

University College London

London

WC1E 6JF

Email: m.ehrenstein@ucl.ac.uk

Keywords: *systemic lupus erythematosus, B cell depletion, Rituximab, Belimumab, randomised clinical trial*

For peer review only

ABSTRACT

Introduction: Few treatment options exist for patients with systemic lupus erythematosus (SLE) who have failed conventional disease modifying drugs. Although widely used to treat moderate to severe lupus, the efficacy of B cell depletion therapy (BCDT) with Rituximab has not been demonstrated in randomised clinical trials. Following BCDT, elevated levels of serum B-cell activating factor (BAFF) have been associated with failure to remit or subsequent lupus relapse. The administration of Belimumab, a monoclonal antibody specific for BAFF and approved for lupus therapy, could potentiate the efficacy of Rituximab and enable longer periods of disease remission. The aim of this trial is to assess the safety and efficacy of Belimumab following Rituximab therapy in patients with SLE.

Methods and analysis: BEAT lupus is a double-blind, randomised, placebo controlled, phase II clinical trial. Patients commencing one cycle of Rituximab (two 1g infusions two weeks apart) as standard of care are randomised to receive Belimumab or placebo, 4 to 8 weeks following the first Rituximab infusion. Belimumab or placebo infusions are administered for 52 weeks. The primary outcome measure is anti-double stranded DNA (dsDNA) antibody levels at 52 weeks. Secondary outcomes include measures of adverse events, lupus disease activity and cumulative steroid dose. The kinetics of B cell repopulation and B and T cell phenotype and function during repopulation will be assessed in a subgroup of participants. Belimumab administration after Rituximab may provide a novel therapeutic pathway for patients with active lupus if safety is demonstrated in this proof of concept study, and lower anti-dsDNA antibodies levels are achieved in those patients on belimumab compared to placebo.

Ethics and Dissemination: Trial information is available at https://www.isrctn.com/ISRCTN47873003, and the results of this trial will be submitted for publication in relevant peer-reviewed publications. Key findings will also be presented at national and international conferences.

For peer review only

Strengths and limitations of this trial

- **Developed as a direct result of experimental data suggesting that flares post-rituximab are driven by elevated BAFF levels. The experimental component of this trial will explore B and T cell kinetics, enabling a greater understanding of response or lack thereof to B cell depletion therapy.**
- **Trial design strictly limits steroid and immunosuppressant dosage.**
- **Small sample size due to safety considerations prevailing at the conception of the trial.**
- **Designed to test whether the UK can recruit sufficiently to a double blind randomised controlled trial in lupus.**
- **Testing safety and efficacy in patients who have failed conventional therapy.**

For peer review only

For peer review only - http://bmjopen.bmj.com/site/about/guidelines.xhtml

Introduction

Despite burgeoning research into the pathogenesis of systemic lupus erythematosus (SLE), the introduction of novel treatments has been slow. This is in contrast to some other immune mediated rheumatic diseases, in particular rheumatoid arthritis (RA), for which several novel therapies developed through increased understanding of disease pathogenesis have made a substantial impact upon patient care. A number of reasons may account for the disparity between SLE and RA including the greater use of corticosteroids which limits the potential for novel treatments to show superiority in clinical trials; the multisystem nature of lupus and insufficient understanding of the key factors that drive disease (1).

Rituximab, a chimeric anti-CD20 monoclonal antibody (mAb), has shown significant, though variable, efficacy in open-label studies, including both single-centre (2-5) and multicentre studies (6-8) (9); as well as a systematic review of off-label use (6). However, two large, phase III, randomised placebo-controlled trials in non-renal lupus (EXPLORER) (10, 11) and renal lupus (LUNAR) (12), failed to meet their primary end-points. Nevertheless, the European League Against Rheumatism (REF) have accepted that therapy with rituximab should be considered in organ-threatening, refractory lupus (13). In addition, NHS England have approved Rituximab as part of standard of care in patients with moderate to severe lupus who have failed conventional immunosuppressant therapy due to the few treatment options available for this patient group (NHS ENGLAND A13/PS/a)(14). However, the variable therapeutic success of rituximab highlights the need to refine therapies that target B cells to improve the outcome for patients with SLE.

The efficiency of B cell depletion following Rituximab treatment has been shown to predict response in patients with lupus (4, 15, 16). Analysis of peripheral B cell subsets revealed that patients with lower plasmablast counts at 6 months are more likely to have a sustained response without requiring retreatment (4). In the subset of SLE patients with high levels of anti-double stranded DNA (anti-dsDNA) antibodies, clinical relapse occurs at lower B cell numbers after treatment compared to before Rituximab and is associated with higher proportions of repopulating plasmablasts (15). Further investigation into the mechanism of Rituximab has demonstrated that levels of the B cell cytokine BAFF (B cell activating factor) surge after B cell depletion (17, 18). Moreover, elevated serum BAFF levels can endure beyond initial B cell repopulation and distinguish lupus relapse from ongoing disease remission following Rituximab (19). In some lupus patients with high levels of anti-dsDNA antibodies, repeated cycles of Rituximab resulted in ever higher levels of serum anti-dsDNA antibodies, which was associated with increasing levels of serum BAFF (16). This effect raises the

For peer review only - http://bmjopen.bmj.com/site/about/guidelines.xhtml

possibility that repeated Rituximab treatments may result in more severe flares driven by BAFF (20). Therefore, high BAFF levels post Rituximab could be limiting its effectiveness in some patients with SLE.

Clinical studies have demonstrated that BAFF is over-expressed, either intermittently or persistently, in many patients with SLE irrespective of Rituximab therapy (21, 22) and serum levels correlate with serologic and clinical indices of disease activity (23). In 2016, the BAFF-neutralizing monoclonal antibody, Belimumab was the first biologic licensed for the treatment of lupus following two large phase III clinical trials, BLISS 52 (24) and BLISS 76 (25). Treatment with Belimumab after B cell depletion has been suggested as a potential therapeutic strategy for patients with SLE (20). We therefore designed a phase II clinical trial to assess the safety and effectiveness of this treatment regime for patients with lupus, as judged by a change in anti-dsDNA antibody levels as the primary outcome. Anti-dsDNA antibodies have been associated with disease activity in patients with SLE (14, 26, 27). Secondary outcomes include reduction in cumulative steroid intake and incidence of disease flares during the 52-week follow-up period. This trial will also provide the opportunity to assess the kinetics of B cell repopulation, and B and T cell phenotype and function over the course of the trial.

For peer review only

Methods and analysis

BEAT lupus is a phase IIb, multicentre, UK based, randomised, double blind, placebo-controlled clinical trial investigating the safety and efficacy of Belimumab (given for 12 months), 4-8 weeks after the first infusion of B cell depletion therapy (Rituximab). Rituximab is administered according to the NHS England interim clinical commissioning policy (NHS England A13/PS/a) and the British Society for Rheumatology guideline for the management of systemic lupus erythematosus in adults (14), specifically two 1 g doses of rituximab two weeks apart. The target population of the trial is patients with a diagnosis of lupus undergoing Rituximab therapy as standard of care. The trial began recruitment in November 2016 and closed for recruitment in March 2019, with data collection of the primary outcome expected to end in March 2020. Collection of safety data will end in April 2020.

Study population

Detailed inclusion and exclusion criteria are listed in Box 1. In brief, patients are aged between 18 and 75 years with 4 or more criteria for SLE, according to the American College of Rheumatology (ACR) 1997 criteria or SLICC 2012 criteria for lupus, or biopsy proven lupus nephritis with one additional supportive test on at least two occasions (positive ANA, anti-dsDNA antibodies or anti-Sm antibodies) (14). Patients had to have a history of anti-dsDNA antibodies detectable at least once in the past 5 years. Participants had to be eligible for treatment with Rituximab as per NHS England Commissioning Policy. A screening and assessment visit are performed before the first (of two) Rituximab infusion, which is 4-8 weeks before randomisation (Day 0). A second screen is performed no more than 10 days before randomisation to confirm eligibility (e.g. excluding patients who required intravenous antibiotics for infections developing after rituximab or low IgG levels, see Box 1). Prior use of Rituximab is allowed, but not within 6 months of initial screening.

For peer review only

BOX 1 Patient inclusion and exclusion criteria
(randomisation to trial (belimumab or placebo occurs at Day 0)
Inclusion criteria
1. Age between 18 and 75 years
2. Participants with 4 or more criteria for SLE according to the American College of Rheumatology (ACR) 1997 criteria or SLICC 2012 criteria or biopsy proven lupus nephritis with one additional supportive test on at least two occasions (positive ANA, anti-dsDNA antibodies or anti-Sm antibodies).
3. History of anti-dsDNA antibodies detectable at least once in the past 5 years prior to screening the participant on the trial protocol (ELISA test is preferable for anti-dsDNA antibody testing).
4. Participants are due to be treated with the first infusion of B cell depletion therapy (Rituximab) 4-8 weeks before randomisation (Day 0). Previous use of Rituximab is allowed prior to this cycle but not within 6 months of screening.
5. No contraindications to the use of Belimumab.
6. Ability to provide informed consent
Exclusion criteria
1. Severe "critical" SLE flare defined as BILAG A flare in CNS system or any SLE manifestation requiring more immunosuppression than allowed within the protocol in the physician's opinion
2. Pregnancy and/or breastfeeding patients
3. At risk of pregnancy and unwilling to use an acceptable form of birth control contraception
4. Prior use of Belimumab, Atacicept or any biologic therapy (except Rituximab, but no other B cell depleting therapies)
5. Participation in any other interventional trial within the last 6 months
6. eGFR <30mls/min at screening
7. Active infections, including but not limited to:
- Current or past infection with hepatitis B or C as defined by:
  Hepatitis B surface antigen positive
  Hepatitis B surface antibody positive and hepatitis B core antibody positive
  Hepatitis C antibody positive
- Historically positive HIV test or test positive at screening for HIV
- Active TB.

8. Infection history:
- Currently on any suppressive therapy for a chronic infection (such as tuberculosis, pneumocystis, cytomegalovirus, herpes simplex virus, herpes zoster and atypical mycobacteria)
- Hospitalization for treatment of infection within 60 days of Day 0
- Use of parenteral (IV or IM) antibiotics (anti-bacterials, antivirals, anti-fungals, or anti-parasitic agents) within 30 days of Day 0
- Receipt of a live-attenuated vaccine within 3 months of Day 0
- In the investigator's opinion, participants that are at high risk for infection (including but not limited to in dwelling catheter, dysphagia with aspiration, decubitus ulcer, history of prior aspiration pneumonia or recurrent severe urinary tract infection)

9. IgG levels below 4.0 g/L, IgA level < 10 mg/dL (IgG and IgA test must be performed no more than 10 days before Day 0
10. Primary immunodeficiency
11. History of malignant neoplasm within the last 5 years
12. History of cervical dysplasia CIN Grade III cervical high-risk human papillomavirus or abnormal cervical cytology other than abnormal squamous cells of undetermined significance (ASCUS) within the past 3 years. The participant will be eligible after the condition has resolved (e.g., follow-up HPV test is negative or cervical abnormality has been effectively treated >1 year ago)
12. Severe, progressive, or uncontrolled renal, hepatic, haematological, gastrointestinal, pulmonary, cardiac, or neurological disease or, in the investigator's opinion, any other concomitant medical condition or significant abnormal laboratory value that places the participant at risk by participating in this trial with the exception of diseases or conditions related to active SLE.
13. Comorbidities not lupus related currently requiring systemic corticosteroid therapy.
14. Evidence of serious suicide risk including any history of suicidal behaviour in the last 6 months and/or any suicidal ideation in the last 2 months or who in the investigator's judgement, pose a significant risk.
15. History of an anaphylactic reaction to parenteral administration of contrast agents, human or murine proteins or monoclonal antibodies.
16. Current drug or alcohol abuse or dependence, or a history of drug or alcohol abuse or dependence within 364 days prior to Day 0.
17. White blood cells (WBC) <1.5 x 10^9/L, Neutrophils <1 x 10^9/L measured up to 10 days before Day 0
18. A history of major organ transplant or haematopoietic stem/cell/marrow transplant or renal transplant.

For peer review only - http://bmjopen.bmj.com/site/about/guidelines.xhtml

Randomisation and Blinding

BEAT Lupus is a double-blind trial. Only the local pharmacist, the unblinded site monitor and the trial statistician will be unblinded to the treatment allocation. All other members of the trial team and patients are blinded to the trial medication throughout the trial. Unblinding will only occur if a participant experiences a serious adverse event for which the clinical management of the SAE will be facilitated by the unblinding of the participant's treatment allocation.

After providing written informed consent, participants are randomised to Belimumab or placebo treatment using a secure online randomisation service provider, Sealed Envelope https://www.sealedenvelope.com/. Randomisation was performed using minimisation, incorporating a random element to ensure balance in the stratifying variables between the two randomised groups, while retaining an element of unpredictability. Stratifying variables are: CD19 count after rituximab at randomisation (<0.01x10^9/l vs >0.01x10^9/l) to account for variability in B cell depletion which would affect response; anti dsDNA (positive or negative at first screen); and whether patients have active renal disease at screening.

Trial treatment

Participants either receive intravenous Belimumab or placebo 4-8 weeks after the first of two infusions of B cell depletion therapy (one cycle Rituximab). The total treatment period (Belimumab or placebo) will be 52 weeks (last infusion of belimumab or placebo at week 52) followed by a safety visit at week 56. In the active therapy arm, participants will receive Belimumab according to the standard dosage regime. The recommended dosage regimen is 10 mg/kg at 2-week intervals for the first 3 doses and at 4-week intervals thereafter. The participants in the placebo group receive the same volume of normal saline infusions (indistinguishable from active treatment) at the same time points as the active treatment group. Participants and physicians are blinded with respect to active treatment or placebo. The assessment schedule is summarised in Figure 1.

Concomitant medications

Before randomisation, which includes the period when Rituximab is administered, treatment is entirely at the physician's discretion, though the rituximab dose is fixed by NHS England (1g administered on two occasions, two weeks apart). All medication is recorded. From the day of randomisation (Day 0, see Figure 1), the maximum dose of Mycophenolate is 1g/day with a suggested dose reduction to 500mg/day at 3 months or as soon as possible thereafter. The

For peer review only - http://bmjopen.bmj.com/site/about/guidelines.xhtml

maximum dose of azathioprine after randomization is 1mg/kg with a suggested dose reduction to 0.5mg/kg by 3 months or as soon as possible thereafter. The maximum dose of methotrexate allowed is 15mg/week with a dose reduction to 10mg/week by 3 months or as soon as possible thereafter. Only one of these three disease modifying drugs (Azathioprine, methotrexate and mycophenolate) can be administered post randomisation. No other immunosuppressants (e.g. cyclophosphamide, tacrolimus) are allowed after randomisation except in the case of severe flare. Background anti-malarial drugs are permitted but no new anti-malarials can be started after randomisation. Mepacrine is acceptable for skin problems resistant to Hydroxychloroquine and both can be combined if this regime was started before randomisation. No change in dose of the anti-malarials can be made after randomisation. Participants will be permitted to receive up to 20 mg prednisolone/day from randomisation. It is strongly encouraged that prednisolone should be reduced by 50% by 6 months post randomisation, with a maximum dose of 5mg/day recommended at 6 months if possible. Participants who flare with one BILAG A or two BILAG B scores (severe flare) will be permitted to receive other therapies or increased prednisolone at the discretion of the treating physician and can also continue with Belimumab or placebo at the discretion of their physician. Centres are encouraged to follow the British Society for Rheumatology guidelines for the management of systemic lupus erythematosus in adults (14).

Trial endpoints

The primary outcome measure is anti-dsDNA antibody levels at 52 weeks. Anti-dsDNA antibodies often correlate with disease activity (14, 26, 27). This biomarker was also chosen due its correlation with BAFF levels in participants post B cell depletion therapy (19). Serum anti-dsDNA antibodies are analysed using the DIASTAT® anti-dsDNA enzyme-linked immunosorbent assay. All samples are tested in the same central lab at UCL.

Secondary outcome measurements include anti-dsDNA antibody levels at 24 weeks and the proportion of participants with serious adverse events. The proportion of participants with a severe disease flare (defined as 1 BILAG A or 2 BILAG B flares) by 24 and 52 weeks following randomisation will be assessed, as will the time to a severe disease flare. Participants who have a severe disease flare will be permitted to receive other therapies or increased prednisolone (including intravenous methylprednisolone/higher doses of Prednisolone/Mycophenolate/Azathioprine) at the discretion of the treating physician and can also continue with Belimumab or placebo at the discretion of their physician. Further cycles of Rituximab are not allowed during the trial even in the event of a severe

For peer review only - http://bmjopen.bmj.com/site/about/guidelines.xhtml

disease flare. Patients who have a severe disease flare will be invited to an additional visit where standard trial data, including serum for the primary endpoint analysis, is collected.

Disease activity will be assessed using the BILAG disease activity index and SLEDAI 2000. C3 and immunoglobulin levels will be recorded. As part of the assessment of response to treatment, we shall calculate the cumulative steroid dose during treatment from randomisation and the proportion of participants decreasing their steroid dose by 50% without flaring from randomisation; or if below 10mg/day at randomisation reducing steroid dose to 5mg/day or who discontinue glucocorticoids with stable disease. Lupus Quality of Life (Lupus QoL), SF-36 and EQ5D will be used as patient reported outcome measures. C-SSRS (Columbia suicide severity rating scale) will be used to assess suicidality risk. The endpoints will be assessed using the outcome measures in Box 2.

For peer review only

BOX 2: OUTCOME MEASURES

Primary outcome measures

Anti-dsDNA-antibody levels at 52 weeks.

Secondary outcome measures

1. Anti-dsDNA antibody levels at 24 weeks
2. Proportion of participants with any adverse events by 52 weeks.
3. Proportion of participants with any serious adverse events by 52 weeks.
4. Proportion of participants with any infections by 52 weeks.
5. Proportion of participants with a severe disease flare (defined as one BILAG A or two B flares) by 52 weeks.
6. Proportion of participants with a severe or a moderate disease flare (defined at least one BILAG B flare accompanied by an increase in concomitant lupus medication - glucocorticoids, Mycophenolate, Azathioprine, or Methotrexate) by 52 weeks.
7. Time to severe disease flare.
8. Proportion of participants with a severe disease flare by 24 weeks.
9. SLEDAI 2000 at 52 weeks.
10. Subject Global Assessment of Disease Activity (SGADA) at 52 weeks.
11. C3 levels at 52 weeks.
12. Immunoglobulin levels at 52 weeks.
13. Cumulative steroid dose from randomisation to 52 weeks.
14. Proportion of participants decreasing steroid dose at randomisation by 50% without experiencing a severe flare, or if below 10mg/day at randomisation reducing dose to 5mg/day or less at 52 weeks.
15. Lupus Quality of Life (Lupus QoL), SF-36 at 52 weeks
16. Average EQ-5D from randomisation to 52 weeks.
17. C-SSRS (Columbian Suicide Severity Rating Scale) to assess suicidality risk at 52 weeks.

Exploratory outcomes

1. BAFF (measurement of RNA from whole blood) at 52 weeks.
2. Kinetics of B cell repopulation, B and T cell phenotype and function during repopulation using flow cytometry.

Trial sample size calculation

We aim to recruit up to 56 SLE participants (the original sample size of 50 was increased to account for a higher than expected dropout rate) and randomise 28 participants to receive Belimumab after Rituximab and 28 participants to receive placebo after Rituximab. The primary outcome is the estimated difference in anti dsDNA binding antibody levels at 12 months, adjusted for anti dsDNA binding levels at screening. Sample size calculations were performed using Stata Release 13 assuming that anti dsDNA binding antibody levels are log normally distributed. The Standard Deviation of anti dsDNA and the correlation structure assumed were based on two sets of data (a)

For peer review only - http://bmjopen.bmj.com/site/about/guidelines.xhtml

the study of 35 participants by Carter et al (19) and (b) unpublished data provided by Prof D Isenberg for 67 participants before and 6 months after B cell depletion therapy (Table 1).

Table 1: Standard deviations and correlations for $\log_e$ anti dsDNA

Dataset	Time point	Log mean	Log sd	Correlation
**Isenberg (n=67)**	**Before rituximab**	6.036	1.393	
	**Follow up**	5.189	1.500	0.612
**Carter (n=35)**	**Before rituximab**	5.091	1.676	
	**Follow up**	5.225	1.781	0.527

On the natural log scale the standard deviation of anti dsDNA at the end of treatment was taken to be 1.7 based on the above data. The correlation between before rituximab and final log anti dsDNA was taken to be 0.55 also based on the above. 22 evaluable participants per group would be able to detect a difference of 1.2 in anti dsDNA binding antibody levels on the natural log scale at the 5% significance level with 80% power. Assuming that 20% of participants fail to attend the 52 week follow-up visit, and taking account of the correlation between screening measurements and 52 week measurements, we aim to recruit 28 participants per group. For example, if we assume that there will be no change in anti dsDNA levels in the Belimumab group, then we would be able to detect an increase of 232% in the placebo group as significantly different. To place this increase in context, Carter et al showed that the difference in anti dsDNA binding antibody levels on the natural log scale between those participants who did and did not have a flare was 1.928, corresponding to a 588% increase in anti dsDNA binding levels in participants with a disease flare (19).

For peer review only

Statistical analysis

A CONSORT diagram will be used to describe the course of participants through the trial. Characteristics at screening and randomisation will be summarised by treatment group.
The analysis of the primary outcome will be done using linear regression to estimate the difference in log anti-dsDNA levels between the two groups at 52 weeks, adjusting for the stratifying variables anti-dsDNA (at screening, pre-Rituximab), BILAG category (renal, non-renal) (at screening, pre-Rituximab), and CD19 count (at randomisation). The analysis will be done on the intention-to-treat

principle; all available 52 week measurements will be included regardless of patient's adherence to their randomised treatment. If withdrawals from follow up before week 52 exceed 10%, a supportive analysis of the primary outcome will be done using multiple imputation (with 52-week anti-dsDNA imputed using baseline and any 12, and 24 weeks measurements where available and other auxiliary information) to evaluate the sensitivity of the results to any differential loss to follow up. Additionally, a supportive sensitivity analysis will be done to estimate the treatment effect for only those patients who adhered to their allocated treatment for 52 weeks, and mediation analyses will be done to evaluate whether the observed effect of Belimumab on anti-dsDNA levels is mediated by differences between groups in the concomitant medication doses (including prednisolone) following randomisation.

A similar modelling approach will be adopted for the analysis of other continuous outcomes measured at screening (before rituximab) and at 52 weeks. Fisher's exact test will be used to compare proportions. Two sample t tests or Mann Whitney U tests, depending on the distribution of the data, will be used to evaluate continuous outcomes compared only at the end of the trial. Kaplan-Meier survival curves will be produced for comparisons of time to an event. The statistical analysis will be performed using Stata.

Ethical considerations

This trial has been approved by the local ethics review board and the MHRA and will be conducted in accordance with ethical principles derived from Good Clinical Practice Guidelines, including the Declaration of Helsinki. Written informed consent will be collected from all patients by approved study site nurse or physician. Patients will be assigned a participant identification number and all patient information transferred to the sponsor will contain this number, patient initials and date of birth. All Serious Adverse Events (SAE) will be reported to the sponsor within 24 hours. Protocol version 5 dated 21st January 2019 is the current protocol version and has been approved.

Dissemination

Trial information is available at https://www.isrctn.com/ISRCTN47873003 including study sites all of which are in England, and the results of this trial will be submitted for publication in relevant peer-reviewed publications. Key findings will also be presented at national and international conferences. Published results will be disseminated to investigators at participating sites, who will further disseminate the results to trial participants on request.

Patient and public involvement

For peer review only - http://bmjopen.bmj.com/site/about/guidelines.xhtml

Feedback gathered from focused patient involvement meetings was used to inform the design of this trial. Patients recognised the need for improving therapies for SLE. They suggested that the inclusion and exclusion criteria should not represent a significant barrier to recruitment and that the trial should be as close to “real-world” practise as possible. Two patient advisors are members of the Trial Steering Committee.

For peer review only

For peer review only - http://bmjopen.bmj.com/site/about/guidelines.xhtml

Discussion

This clinical trial has been developed through investigation and analysis of clinical and laboratory data of lupus patients receiving Rituximab. In particular, we noted increased serum BAFF levels correlated with a rise in anti-DNA antibodies at the time of flare in lupus patients post Rituximab (19). We therefore predicted that lupus patients treated with Belimumab following Rituximab therapy will have lower anti-dsDNA antibody levels compared to those that have received Rituximab alone, and that this combination would have greater efficacy than Rituximab alone. Given the paucity of novel therapies, and the many clinical trials that have failed to show efficacy for SLE, a combination of biologic therapies with complementary effects given in succession may be required to control disease. If the results of this trial are promising, a larger trial will be required to achieve sufficient power to detect improved clinical outcomes. Indeed, a larger trial is already underway (BLISS BELIEVE NCT03312907) testing whether rituximab given after belimumab confers an additional benefit compared to belimumab alone (28). The order of administration of these two biologics is reversed compared to BEAT-LUPUS and there is no Rituximab alone arm. The administration of Rituximab first allows for the detection of adverse events that occur in the 4-8 week period after Rituximab and before randomisation. A second screen 7-10 days before randomisation will exclude patients based on these adverse events. CALIBRATE (NCT02260934), a clinical trial for renal lupus uses a similar sequence of these two biologics to the trial reported here, though cyclophosphamide is administered with the rituximab, and higher doses of concomitant medications are allowed.

Recruitment to lupus trials has been challenging in the UK, and few academic led interventional SLE trials in the UK have met their recruitment target. The last academic led lupus trial in the UK not subject to early termination because of poor recruitment commenced in 1997 and completed recruitment in 2005 (though even this open label trial's recruitment target had to be reduced due to slow recruitment) (29). We endeavoured to optimise recruitment by designing a protocol that would be as close to real life clinical practice as possible. Consequently, there are no therapeutic restrictions before patients are randomised (to receive either Belimumab or placebo) giving therapeutic freedom to the treating physician including the possibility of administering high dose corticosteroids in conjunction with Rituximab. Rituximab had to be administered 4-8 weeks before randomisation and is administered in accordance with the NHS England's Interim Commissioning Policy (NHS ENGLAND A13/PS/a) for patients with refractory lupus (14). This policy only allows Rituximab to be given to patients who have failed to respond adequately to either mycophenolate or cyclophosphamide, unless contraindicated, or requiring unacceptably high doses of corticosteroids. Thus, the results of this trial will be relevant to lupus patients that are refractory to or intolerant of

For peer review only - http://bmjopen.bmj.com/site/about/guidelines.xhtml

conventional therapy, the patient group for which this combination therapy is most likely to be approved. The trial provides information about rates of recruitment to lupus trials in England which could inform the development and feasibility of other UK based trials planned in the future. Recruitment was aided through NHS England's policy applicable to biological therapies for SLE which encourages physicians and their patients to consider interventional clinical trials (NHS ENGLAND A13/PS/a) (14).

The trial protocol offers physicians some flexibility with the use of concomitant immunosuppressant and oral prednisolone after randomisation. However, because of safety concerns, and to maximise the chances of revealing any potential difference between the two treatment arms, corticosteroids are limited to a maximum dose of 20mg/day at randomisation and it is strongly encouraged to taper the dose by at least 50% by 6 months. Moreover, immunosuppressant doses are also restricted. It can be difficult to achieve reductions in both corticosteroids and immunosuppressants in patients who, by the nature of the inclusion criteria, are more resistant to therapy. Our strategy has been to advise that reduction in corticosteroid dose should be prioritised over tapering immunosuppressants due to the former's more problematic side effects, and its ability to mask the difference between treatment arms in lupus trials.

This trial has some limitations. Firstly, the sample size is relatively small because at the time of the trial's inception there were no published safety data on the combination of Rituximab and Belimumab and an earlier phase, smaller trial was advised. Therefore, the trial is powered on anti-DNA antibodies rather than a clinical endpoint. To limit the risk of adverse events due to immunosuppression, the dose of concomitant DMARDs is lower than routinely prescribed for patients with active disease, particularly renal patients receiving mycophenolate. This may have reduced recruitment of patients with active nephritis by some physicians, although this restriction in dosage could also be an advantage with respect to distinguishing between active drug and placebo. The serological inclusion criteria enable patients with a confirmed positive dsDNA antibody within the last 5 years to participate in the trial rather than patients who have raised anti-DNA antibodies at screen. This approach was taken to maximise recruitment but also with the recognition that flares post Rituximab can, in a proportion of patients, be associated with increased DNA antibody levels above that observed when Rituximab is first commenced (15).

The development of this trial has arisen directly from analysis of the immunologic changes following B cell depletion therapy. Building on this experimental medicine approach, analysis of B and T cell phenotype has been incorporated into this trial to understand how sequential targeting of B cells

For peer review only - http://bmjopen.bmj.com/site/about/guidelines.xhtml

and BAFF affect B cell numbers and phenotype. An exploratory analysis will aim to determine if elevated serum autoantibodies and BAFF, present at screening (before rituximab) or at randomisation, can predict an improved response to Belimumab after Rituximab. If the overall results from this trial are encouraging, a larger definitive trial using time to flare as the primary outcome may be considered after taking into account the results from other similar trials currently underway. The increasing interest in this combination therapy has the potential to offer a novel treatment option for patients with active lupus.

Funding

This trial is supported by Versus Arthritis (grant number 20873) and the UCLH Biomedical Research Centre (BRC). GSK are providing Belimumab free of charge, as well as additional funding. The MRC (MASTERPLANS CONSORTIUM) is supporting some of the experimental medicine applied to samples from this trial.

Conflict of interest statement

MRE has received grant/research support from GSK. CG has been a member of the speakers bureau for GSK and has received consultancy fees for attending advisory boards.

Contributorship

MRE conceived of the study, MRE, CG, CD and DAI initiated the study design. PM and KC and CD provided statistical expertise in clinical trial in clinical trial design. AJ, MRE, CG, CD, FI and EC assisted with protocol development. AJ, MRE, PM, CD wrote the manuscript, all authors reviewed the manuscript. MRE, DAI, CG and CD are grant holders.

Acknowledgements

We acknowledge the important contribution of the BEAT Lupus Trial Steering Committee, NIHR, the Data Monitoring Committee, and all the patients participating in the trial or involved in its development. All authors reviewed and edited the draft version of the manuscript and approved the final version. All authors have approved the final manuscript submitted. We are indebted to the BEAT-LUPUS Trial Collaborators at the recruiting sites and British Isles Lupus Assessment Group (BILAG). The trial is sponsored by University College London, Gower Street London, WC1E 6BT, United Kingdom, +44 20 7679 6163, ctu.beatlupus@ucl.ac.uk.

Trial registration number: ISRCTN47873

https://www.isrctn.com/ISRCTN47873003

For peer review only - http://bmjopen.bmj.com/site/about/guidelines.xhtml

REFERENCES

1. Isenberg DA, Merrill JT. Why, why, why de-lupus (does so badly in clinical trials). Expert Rev Clin Immunol. 2016;12(2):95-8.

2. Galarza-Maldonado C, Kourilovitch MR, Molineros JE, Cardiel MH, Zurita L, Soroka NF, et al. The administration of low doses of rituximab followed by hydroxychloroquine, prednisone and low doses of mycophenolate mofetil is an effective therapy in Latin American patients with active systemic lupus erythematosus. Autoimmunity reviews. 2010;10(2):108-11.

3. Leandro MJ, Edwards JC, Cambridge G, Ehrenstein MR, Isenberg DA. An open study of B lymphocyte depletion in systemic lupus erythematosus. Arthritis & Rheumatism. 2002;46(10):2673-7.

4. Vital EM, Dass S, Buch MH, Henshaw K, Pease CT, Martin MF, et al. B cell biomarkers of rituximab responses in systemic lupus erythematosus. Arthritis & Rheumatism. 2011;63(10):3038-47.

5. Aguiar R, Araújo C, Martins-Coelho G, Isenberg D. Use of Rituximab in Systemic Lupus Erythematosus: A Single Center Experience Over 14 Years. Arthritis Care & Research. 2017;69(2):257-62.

6. Ramos-Casals M, Brito-Zerón P, Muñoz S, Soto M-J. A systematic review of the off-label use of biological therapies in systemic autoimmune diseases. Medicine. 2008;87(6):345-64.

7. Díaz-Lagares C, Croca S, Sangle S, Vital EM, Catapano F, Martínez-Berriotxoa A, et al. Efficacy of rituximab in 164 patients with biopsy-proven lupus nephritis: pooled data from European cohorts. Autoimmunity reviews. 2012;11(5):357-64.

8. Witt M, Grunke M, Proft F, Baeuerle M, Aringer M, Burmester G, et al. Clinical outcomes and safety of rituximab treatment for patients with systemic lupus erythematosus (SLE)–results from a nationwide cohort in Germany (GRAID). Lupus. 2013;22(11):1142-9.

9. McCarthy EM, Sutton E, Nesbit S, White J, Parker B, Jayne D, et al. Short-term efficacy and safety of rituximab therapy in refractory systemic lupus erythematosus: results from the British Isles Lupus Assessment Group Biologics Register. Rheumatology. 2017;57(3):470-9.

10. Merrill J, Buyon J, Furie R, Latinis K, Gordon C, Hsieh H, et al. Assessment of flares in lupus patients enrolled in a phase II/III study of rituximab (EXPLORER). Lupus. 2011;20(7):709-16.

11. Merrill JT, Neuwelt CM, Wallace DJ, Shanahan JC, Latinis KM, Oates JC, et al. Efficacy and safety of rituximab in moderately-to-severely active systemic lupus erythematosus: the randomized, double-blind, phase II/III systemic lupus erythematosus evaluation of rituximab trial. Arthritis & Rheumatism: Official Journal of the American College of Rheumatology. 2010;62(1):222-33.

12. Rovin BH, Furie R, Latinis K, Looney RJ, Fervenza FC, Sanchez-Guerrero J, et al. Efficacy and safety of rituximab in patients with active proliferative lupus nephritis: the Lupus Nephritis Assessment with Rituximab study. Arthritis & Rheumatism. 2012;64(4):1215-26.

13. Fanouriakis A, Kostopoulou M, Alunno A, Aringer M, Bajema I, Boletis JN, et al. 2019 update of the EULAR recommendations for the management of systemic lupus erythematosus. Annals of the Rheumatic Diseases. 2019;78(6):736-45.

14. Gordon C, Amissah-Arthur M-B, Gayed M, Brown S, Bruce IN, D'Cruz D, et al. The British Society for Rheumatology guideline for the management of systemic lupus erythematosus in adults. Rheumatology. 2017;57(1):e1-e45.

For peer review only - http://bmjopen.bmj.com/site/about/guidelines.xhtml

15. Lazarus MN, Turner-Stokes T, Chavele K-M, Isenberg DA, Ehrenstein MR. B-cell numbers and phenotype at clinical relapse following rituximab therapy differ in SLE patients according to anti-dsDNA antibody levels. Rheumatology. 2012;51(7):1208-15.
16. Iaccarino L, Bartoloni E, Carli L, Ceccarelli F, Conti F, De SV, et al. Efficacy and safety of off-label use of rituximab in refractory lupus: data from the Italian Multicentre Registry. Clinical and experimental rheumatology. 2015;33(4):449-56.
17. Cambridge G, Isenberg DA, Edwards J, Leandro MJ, Migone T-S, Teodorescu M, et al. B cell depletion therapy in systemic lupus erythaematosus: relationships among serum B lymphocyte stimulator levels, autoantibody profile and clinical response. Annals of the rheumatic diseases. 2008;67(7):1011-6.
18. Vallerskog T, Heimbürger M, Gunnarsson I, Zhou W, Wahren-Herlenius M, Trollmo C, et al. Differential effects on BAFF and APRIL levels in rituximab-treated patients with systemic lupus erythematosus and rheumatoid arthritis. Arthritis research & therapy. 2006;8(6):R167.
19. Carter LM, Isenberg DA, Ehrenstein MR. Elevated Serum BAFF Levels Are Associated With Rising Anti–Double-Stranded DNA Antibody Levels and Disease Flare Following B Cell Depletion Therapy in Systemic Lupus Erythematosus. Arthritis & Rheumatism. 2013;65(10):2672-9.
20. Ehrenstein MR, Wing C. The BAFFling effects of rituximab in lupus: danger ahead? Nature Reviews Rheumatology. 2016;12(6):367.
21. Cheema GS, Roschke V, Hilbert DM, Stohl W. Elevated serum B lymphocyte stimulator levels in patients with systemic immune–based rheumatic diseases. Arthritis & Rheumatism: Official Journal of the American College of Rheumatology. 2001;44(6):1313-9.
22. Stohl W, Metyas S, Tan SM, Cheema GS, Oamar B, Xu D, et al. B lymphocyte stimulator overexpression in patients with systemic lupus erythematosus: longitudinal observations. Arthritis & Rheumatism: Official Journal of the American College of Rheumatology. 2003;48(12):3475-86.
23. Petri M, Stohl W, Chatham W, McCune WJ, Chevrier M, Ryel J, et al. Association of plasma B lymphocyte stimulator levels and disease activity in systemic lupus erythematosus. Arthritis & Rheumatism: Official Journal of the American College of Rheumatology. 2008;58(8):2453-9.
24. Navarra SV, Guzmán RM, Gallacher AE, Hall S, Levy RA, Jimenez RE, et al. Efficacy and safety of belimumab in patients with active systemic lupus erythematosus: a randomised, placebo-controlled, phase 3 trial. The Lancet. 2011;377(9767):721-31.
25. Furie R, Petri M, Zamani O, Cervera R, Wallace DJ, Tegzová D, et al. A phase III, randomized, placebo-controlled study of belimumab, a monoclonal antibody that inhibits B lymphocyte stimulator, in patients with systemic lupus erythematosus. Arthritis & Rheumatism. 2011;63(12):3918-30.
26. Isenberg D, Manson J, Ehrenstein M, Rahman A. Fifty years of anti-ds DNA antibodies: are we approaching journey's end? 2007.
27. Floris A, Piga M, Cauli A, Mathieu A. Predictors of flares in Systemic Lupus Erythematosus: Preventive therapeutic intervention based on serial anti-dsDNA antibodies assessment. Analysis of a monocentric cohort and literature review. Autoimmunity Reviews. 2016;15(7):656-63.
28. Teng YKO, Bruce IN, Diamond B, Furie RA, van Vollenhoven RF, Gordon D, et al. Phase III, multicentre, randomised, double-blind, placebo-controlled, 104-week study of subcutaneous belimumab administered in combination with rituximab in adults with systemic lupus erythematosus (SLE): BLISS-BELIEVE study protocol. BMJ Open. 2019;9(3):e025687.

For peer review only - http://bmjopen.bmj.com/site/about/guidelines.xhtml

29. Griffiths B, Emery P, Ryan V, Isenberg D, Akil M, Thompson R, et al. The BILAG multi-centre open randomized controlled trial comparing ciclosporin vs azathioprine in patients with severe SLE. Rheumatology. 2010;49(4):723-32.

For peer review only

For peer review only - http://bmjopen.bmj.com/site/about/guidelines.xhtml

STANDARD PROTOCOL ITEMS: RECOMMENDATIONS FOR INTERVENTIONAL TRIALS

SPIRIT 2013 Checklist: Recommended items to address in a clinical trial protocol and related documents*

Section/item	Item No	Description	Page Number on which item is reported
**Administrative information**			
Title	1	Descriptive title identifying the study design, population, interventions, and, if applicable, trial acronym	1
Trial registration	2a	Trial identifier and registry name. If not yet registered, name of intended registry	2
	2b	All items from the World Health Organization Trial Registration Data Set	
Protocol version	3	Date and version identifier	9
Funding	4	Sources and types of financial, material, and other support	12
Roles and responsibilitie s	5a	Names, affiliations, and roles of protocol contributors	12
	5b	Name and contact information for the trial sponsor	12
	5c	Role of study sponsor and funders, if any, in study design; collection, management, analysis, and interpretation of data; writing of the report; and the decision to submit the report for publication, including whether they will have ultimate authority over any of these activities	
	5d	Composition, roles, and responsibilities of the coordinating centre, steering committee, endpoint adjudication committee, data management team, and other individuals or groups overseeing the trial, if applicable (see Item 21a for data monitoring committee)	
**Introduction**			

For peer review only

Background and rationale	6a	Description of research question and justification for undertaking the trial, including summary of relevant studies (published and unpublished) examining benefits and harms for each intervention	4
	6b	Explanation for choice of comparators	4-5
Objectives	7	Specific objectives or hypotheses	2
Trial design	8	Description of trial design including type of trial (eg, parallel group, crossover, factorial, single group), allocation ratio, and framework (eg, superiority, equivalence, noninferiority, exploratory)	6-10
**Methods: Participants, interventions, and outcomes**			
Study setting	9	Description of study settings (eg, community clinic, academic hospital) and list of countries where data will be collected. Reference to where list of study sites can be obtained	10
Eligibility criteria	10	Inclusion and exclusion criteria for participants. If applicable, eligibility criteria for study centres and individuals who will perform the interventions (eg, surgeons, psychotherapists)	14
Interventions	11a	Interventions for each group with sufficient detail to allow replication, including how and when they will be administered	7
	11b	Criteria for discontinuing or modifying allocated interventions for a given trial participant (eg, drug dose change in response to harms, participant request, or improving/worsening disease)	7
	11c	Strategies to improve adherence to intervention protocols, and any procedures for monitoring adherence (eg, drug tablet return, laboratory tests)	
	11d	Relevant concomitant care and interventions that are permitted or prohibited during the trial	7
Outcomes	12	Primary, secondary, and other outcomes, including the specific measurement variable (eg, systolic blood pressure), analysis metric (eg, change from baseline, final value, time to event), method of aggregation (eg, median, proportion), and time point for each outcome. Explanation of the clinical relevance of chosen efficacy and harm outcomes is strongly recommended	15

For peer review only - http://bmjopen.bmj.com/site/about/guidelines.xhtml

Participant timeline	13	Time schedule of enrolment, interventions (including any run-ins and washouts), assessments, and visits for participants. A schematic diagram is highly recommended (see Figure)	16, Figure 1
Sample size	14	Estimated number of participants needed to achieve study objectives and how it was determined, including clinical and statistical assumptions supporting any sample size calculations	8
Recruitment	15	Strategies for achieving adequate participant enrolment to reach target sample size	
**Methods: Assignment of interventions (for controlled trials)**			
Allocation:			
Sequence generation	16a	Method of generating the allocation sequence (eg, computer-generated random numbers), and list of any factors for stratification. To reduce predictability of a random sequence, details of any planned restriction (eg, blocking) should be provided in a separate document that is unavailable to those who enrol participants or assign interventions	6
Allocation concealment mechanism	16b	Mechanism of implementing the allocation sequence (eg, central telephone; sequentially numbered, opaque, sealed envelopes), describing any steps to conceal the sequence until interventions are assigned	
Implementation	16c	Who will generate the allocation sequence, who will enrol participants, and who will assign participants to interventions	
Blinding (masking)	17a	Who will be blinded after assignment to interventions (eg, trial participants, care providers, outcome assessors, data analysts), and how	6
	17b	If blinded, circumstances under which unblinding is permissible, and procedure for revealing a participant's allocated intervention during the trial	6
**Methods: Data collection, management, and analysis**			

Data collection methods	18a	Plans for assessment and collection of outcome, baseline, and other trial data, including any related processes to promote data quality (eg, duplicate measurements, training of assessors) and a description of study instruments (eg, questionnaires, laboratory tests) along with their reliability and validity, if known. Reference to where data collection forms can be found, if not in the protocol	
	18b	Plans to promote participant retention and complete follow-up, including list of any outcome data to be collected for participants who discontinue or deviate from intervention protocols	
Data management	19	Plans for data entry, coding, security, and storage, including any related processes to promote data quality (eg, double data entry; range checks for data values). Reference to where details of data management procedures can be found, if not in the protocol	
Statistical methods	20a	Statistical methods for analysing primary and secondary outcomes. Reference to where other details of the statistical analysis plan can be found, if not in the protocol	9
	20b	Methods for any additional analyses (eg, subgroup and adjusted analyses)	
	20c	Definition of analysis population relating to protocol non-adherence (eg, as randomised analysis), and any statistical methods to handle missing data (eg, multiple imputation)	
**Methods: Monitoring**			
Data monitoring	21a	Composition of data monitoring committee (DMC); summary of its role and reporting structure; statement of whether it is independent from the sponsor and competing interests; and reference to where further details about its charter can be found, if not in the protocol. Alternatively, an explanation of why a DMC is not needed	
	21b	Description of any interim analyses and stopping guidelines, including who will have access to these interim results and make the final decision to terminate the trial	

For peer review only - http://bmjopen.bmj.com/site/about/guidelines.xhtml

Harms	22	Plans for collecting, assessing, reporting, and managing solicited and spontaneously reported adverse events and other unintended effects of trial interventions or trial conduct	
Auditing	23	Frequency and procedures for auditing trial conduct, if any, and whether the process will be independent from investigators and the sponsor	
**Ethics and dissemination**			
Research ethics approval	24	Plans for seeking research ethics committee/institutional review board (REC/IRB) approval	9
Protocol amendments	25	Plans for communicating important protocol modifications (eg, changes to eligibility criteria, outcomes, analyses) to relevant parties (eg, investigators, REC/IRBs, trial participants, trial registries, journals, regulators)	
Consent or assent	26a	Who will obtain informed consent or assent from potential trial participants or authorised surrogates, and how (see Item 32)	9
	26b	Additional consent provisions for collection and use of participant data and biological specimens in ancillary studies, if applicable	
Confidentiality	27	How personal information about potential and enrolled participants will be collected, shared, and maintained in order to protect confidentiality before, during, and after the trial	
Declaration of interests	28	Financial and other competing interests for principal investigators for the overall trial and each study site	
Access to data	29	Statement of who will have access to the final trial dataset, and disclosure of contractual agreements that limit such access for investigators	
Ancillary and post-trial care	30	Provisions, if any, for ancillary and post-trial care, and for compensation to those who suffer harm from trial participation	
Dissemination policy	31a	Plans for investigators and sponsor to communicate trial results to participants, healthcare professionals, the public, and other relevant groups (eg, via publication, reporting in results databases, or other data sharing arrangements), including any publication restrictions	10

For peer review only

	31b	Authorship eligibility guidelines and any intended use of professional writers	
	31c	Plans, if any, for granting public access to the full protocol, participant-level dataset, and statistical code	
**Appendices**			
Informed consent materials	32	Model consent form and other related documentation given to participants and authorised surrogates	
Biological specimens	33	Plans for collection, laboratory evaluation, and storage of biological specimens for genetic or molecular analysis in the current trial and for future use in ancillary studies, if applicable	

*It is strongly recommended that this checklist be read in conjunction with the SPIRIT 2013 Explanation & Elaboration for important clarification on the items. Amendments to the protocol should be tracked and dated. The SPIRIT checklist is copyrighted by the SPIRIT Group under the Creative Commons "Attribution-NonCommercial-NoDerivs 3.0 Unported" license.

For peer review only

BMJ Open

Belimumab after B cell depletion therapy in patients with systemic lupus erythematosus (BEAT LUPUS) protocol: A prospective multi-centre, double-blind, randomised, placebo-controlled, 52-week phase II clinical trial

Journal:	*BMJ Open*
Manuscript ID	bmjopen-2019-032569.R2
Article Type:	Protocol
Date Submitted by the Author:	21-Nov-2019
Complete List of Authors:	Jones, Alexis; University College London, Division of Medicine<br>Muller, Patrick; University College London, Comprehensive Clinical Trials Unit<br>Dore, Caroline; MRC Clinical Trials Unit,<br>Ikeji, Felicia; University College London, Comprehensive Clinical Trials Unit<br>Caverly, Emilia; University College London, Comprehensive Clinical Trials Unit<br>Chowdhury, Kashfia; University College London, Comprehensive Clinical Trials Unit<br>Isenberg, David; Centre for Rheumatology Research, Division of Medicine UCL , Division of Medicine, UCL<br>Gordon, Caroline; Birmingham University Medical School, Rheumatology Research Group<br>Ehrenstein, MR; University College London, Division of Medicine
**Primary Subject Heading**:	Rheumatology
Secondary Subject Heading:	Immunology (including allergy)
Keywords:	RHEUMATOLOGY, Rheumatology < INTERNAL MEDICINE, IMMUNOLOGY

SCHOLARONE™
Manuscripts

Belimumab after B cell depletion therapy in patients with systemic lupus erythematosus (BEAT LUPUS) protocol: A prospective multi-centre, double-blind, randomised, placebo-controlled, 52-week phase II clinical trial

Authors:

Alexis Jones[1], Patrick Muller[2], Caroline J Doré[2], Felicia Ikeji[2], Emilia Caverly[2], Kashfia Chowdhury[2], David A Isenberg[1], Caroline Gordon[3], and Michael R Ehrenstein[1].

[1] Department of Rheumatology, University College London Hospital, London, UK

[2] Comprehensive Clinical Trials Unit, University College London, London, UK

[3] Rheumatology Research Team - Inflammation and Ageing (IIA), University of Birmingham Research Laboratories, New Queen Elizabeth Hospital, Birmingham, UK

Correspondence to

Michael Ehrenstein

Department of Rheumatology

University College London

London

WC1E 6JF

Email: m.ehrenstein@ucl.ac.uk

Keywords: *systemic lupus erythematosus, B cell depletion, rituximab, belimumab, randomised clinical trial*

For peer review only

ABSTRACT

Introduction: Few treatment options exist for patients with systemic lupus erythematosus (SLE) who fail conventional therapy. Although widely used to treat lupus, the efficacy of B cell depletion therapy using rituximab has not been demonstrated in randomised clinical trials. Following rituximab, elevated levels of serum B-cell activating factor (BAFF) have been associated with failure to remit or subsequent lupus relapse. The administration of belimumab, a monoclonal antibody specific for BAFF and approved for lupus therapy, could potentiate the efficacy of rituximab and enable longer periods of disease remission. The aim of this trial is to assess the safety and efficacy of belimumab following rituximab in patients with SLE.

Methods and analysis: BEAT lupus is a double-blind, randomised, placebo controlled, phase II clinical trial. Patients with SLE commencing one cycle of rituximab (two 1g infusions two weeks apart) as standard of care will be randomised to receive belimumab or placebo, 4 to 8 weeks following the first rituximab infusion. Belimumab or placebo infusions are administered for 52 weeks. The primary outcome measure is anti-double stranded DNA (anti-dsDNA) antibody levels at 52 weeks. Secondary outcomes include measures of adverse events, lupus disease activity and cumulative steroid dose. The kinetics of B cell repopulation will be assessed in a subgroup of participants. Belimumab administration after rituximab may provide a novel therapeutic pathway for patients with active lupus if safety is demonstrated in this proof of concept study, and lower anti-dsDNA antibodies levels are achieved in those patients treated with belimumab compared to placebo.

Ethics and Dissemination: The protocol has been reviewed and approved by the Hampstead Research Ethics Committee-London (reference 16/LO/1024). Trial information is available at https://www.isrctn.com/ISRCTN47873003, and the results of this trial will be submitted for publication in relevant peer-reviewed journals. Key findings will also be presented at national and international conferences.

For peer review only

For peer review only - http://bmjopen.bmj.com/site/about/guidelines.xhtml

Strengths and limitations of this study

- **Double-blind, randomised, placebo controlled trial in lupus patients refractory to conventional therapy thereby reflecting real world practise whilst eliminating expectation of treatment benefit.**
- **Trial recruitment at point of randomisation to belimumab or placebo, rather than before rituximab, to improve safety and mitigate loss of patients due to adverse events following rituximab.**
- **Small sample size because of safety considerations prevailing at the conception of the trial may limit analyses.**
- **Trial design stipulates reduction in concomitant steroid and immunosuppressant dosage to maximise any potential difference between belimumab and placebo.**
- **The experimental medicine component of this trial will enable a greater understanding of response to combination B cell depletion and belimumab therapy.**

For peer review only

For peer review only - http://bmjopen.bmj.com/site/about/guidelines.xhtml

Introduction

Despite burgeoning research into the pathogenesis of systemic lupus erythematosus (SLE), the introduction of novel treatments into the clinic has been slow. This is in contrast to some other immune mediated rheumatic diseases, in particular rheumatoid arthritis (RA), for which several novel therapies developed through increased understanding of disease pathogenesis have made a substantial impact upon patient care. A number of reasons may account for the disparity between SLE and RA including the greater use of corticosteroids which limits the potential for novel treatments to show superiority in clinical trials; the multisystem nature of lupus and insufficient understanding of the key factors that drive disease (1).

Rituximab, a chimeric anti-CD20 monoclonal antibody, has shown significant, though variable, efficacy in open-label studies, including both single-centre (2-5) and multicentre studies (6-9); as well as a systematic review of off-label use (6). However, two large, phase III, randomised placebo-controlled trials in non-renal lupus (EXPLORER) (10, 11) and renal lupus (LUNAR) (12), failed to meet their primary end-points. Nevertheless, the European League Against Rheumatism have accepted that therapy with rituximab should be considered in organ-threatening, refractory lupus (13). In addition, NHS England have approved rituximab as part of standard of care in patients with moderate to severe lupus who have failed conventional immunosuppressant therapy due to the few treatment options available for this patient group (NHS ENGLAND A13/PS/a)(14). However, the variable therapeutic success of rituximab highlights the need to refine therapies that target B cells to improve the outcome for patients with SLE.

The efficiency of B cell depletion following rituximab treatment has been shown to predict response in patients with lupus (4, 15, 16). Analysis of peripheral B cell subsets revealed that patients with lower plasmablast counts at 6 months are more likely to have a sustained response without requiring retreatment (4). In the subset of SLE patients with high levels of anti-double stranded DNA (anti-dsDNA) antibodies, clinical relapse occurs at lower B cell numbers after treatment compared to before rituximab and is associated with higher proportions of repopulating plasmablasts in the peripheral blood (15). Further investigation into the mechanism of rituximab has demonstrated that levels of the B cell cytokine BAFF (B cell activating factor) surge after B cell depletion (17, 18). Moreover, elevated serum BAFF levels can endure beyond initial B cell repopulation and distinguish lupus relapse from ongoing disease remission following rituximab (19). In some lupus patients repeated cycles of rituximab resulted in ever higher levels of serum anti-dsDNA antibodies, which were associated with increasing levels of serum BAFF (16). This observation raises the possibility that

For peer review only - http://bmjopen.bmj.com/site/about/guidelines.xhtml

repeated rituximab treatments may result in more severe flares driven by BAFF (20). Thus, high BAFF levels post rituximab could be limiting its effectiveness in some patients with SLE.

Clinical studies have demonstrated that BAFF is over-expressed, either intermittently or persistently, in many patients with SLE irrespective of rituximab therapy (21, 22) and serum levels correlate with serologic and clinical indices of disease activity (23). In 2016, the BAFF-neutralizing monoclonal antibody, belimumab was the first biologic licensed for the treatment of lupus following two large phase III clinical trials, BLISS 52 (24) and BLISS 76 (25). Treatment with belimumab after B cell depletion has been suggested as a potential therapeutic strategy for patients with SLE (20). We therefore designed a phase II clinical trial to assess the safety and effectiveness of this treatment regime for patients with lupus, the latter judged by a change in anti-dsDNA antibody levels as the primary outcome. Anti-dsDNA antibodies have been associated with disease activity in patients with SLE (14, 26, 27). Secondary outcomes include incidence of adverse events, reduction in cumulative steroid intake and incidence of disease flares during the 52-week follow-up period. This trial will also assess the kinetics of B cell repopulation over the course of the trial.

For peer review only

Methods and analysis

BEAT lupus is a phase IIb, multicentre, UK based, randomised, double blind, placebo-controlled interventional clinical trial investigating the safety and efficacy of belimumab (given for 12 months), 4-8 weeks after the first infusion of B cell depletion therapy (rituximab). Rituximab is administered according to the NHS England interim clinical commissioning policy (NHS England A13/PS/a) and the British Society for Rheumatology guideline for the management of systemic lupus erythematosus in adults (14), specifically two 1 g doses of rituximab given intravenously two weeks apart. The target population of the trial is patients with a diagnosis of lupus undergoing rituximab therapy as standard of care. The trial began recruitment in November 2016 and closed for recruitment in March 2019, all patients were recruited in England, with data collection of the primary outcome expected to end in March 2020. Collection of safety data will end in April 2020.

Study population

Detailed inclusion and exclusion criteria are listed in Box 1. In brief, patients are aged between 18 and 75 years with 4 or more criteria for SLE, according to the American College of Rheumatology (ACR) 1997 criteria or SLICC 2012 criteria for lupus, or biopsy proven lupus nephritis with one additional supportive test on at least two occasions (positive ANA, anti-dsDNA antibodies or anti-Sm antibodies) (14). Patients had to have a history of anti-dsDNA antibodies detectable at least once in the past 5 years. Participants had to be eligible for treatment with rituximab as per NHS England Commissioning Policy. A screening and assessment visit are performed before the first (of two) rituximab infusion, which is 4-8 weeks before randomisation (Day 0, Figure 1). A second screen is performed no more than 10 days before randomisation to confirm eligibility (e.g. excluding patients who required intravenous antibiotics for infections developing after rituximab or low IgG levels, see Box 1). Prior use of rituximab is allowed, but not within 6 months of initial screening.

For peer review only - http://bmjopen.bmj.com/site/about/guidelines.xhtml

BOX 1 Participant inclusion and exclusion criteria
(randomisation to trial (belimumab or placebo occurs at Day 0)
Inclusion criteria

1. Age between 18 and 75 years
2. Participants with 4 or more criteria for SLE according to the American College of Rheumatology (ACR) 1997 criteria or SLICC 2012 criteria or biopsy proven lupus nephritis with one additional supportive test on at least two occasions (positive ANA, anti-dsDNA antibodies or anti-Sm antibodies).
3. History of anti-dsDNA antibodies detectable at least once in the past 5 years prior to screening the participant on the trial protocol (ELISA test is preferable for anti-dsDNA antibody testing).
4. Participants are due to be treated with the first infusion of B cell depletion therapy (Rituximab) 4-8 weeks before randomisation (Day 0). Previous use of Rituximab is allowed prior to this cycle but not within 6 months of screening.
5. No contraindications to the use of Belimumab.
6. Ability to provide informed consent

Exclusion criteria

1. Severe "critical" SLE flare defined as BILAG A flare in CNS system or any SLE manifestation requiring more immunosuppression than allowed within the protocol in the physician's opinion
2. Pregnancy and/or breastfeeding patients
3. At risk of pregnancy and unwilling to use an acceptable form of birth control contraception
4. Prior use of Belimumab, Atacicept or any biologic therapy (except Rituximab, but no other B cell depleting therapies)
5. Participation in any other interventional trial within the last 6 months
6. eGFR <30mls/min at screening
7. Active infections, including but not limited to:
   - Current or past infection with hepatitis B or C as defined by:

Hepatitis B surface antigen positive
Hepatitis B surface antibody positive and hepatitis B core antibody positive
Hepatitis C antibody positive

   - Historically positive HIV test or test positive at screening for HIV
   - Active TB.

8. Infection history:
   - Currently on any suppressive therapy for a chronic infection (such as tuberculosis, pneumocystis, cytomegalovirus, herpes simplex virus, herpes zoster and atypical mycobacteria)
   - Hospitalization for treatment of infection within 60 days of Day 0
   - Use of parenteral (IV or IM) antibiotics (anti-bacterials, antivirals, anti-fungals, or anti-parasitic agents) within 30 days of Day 0
   - Receipt of a live-attenuated vaccine within 3 months of Day 0
   - In the investigator's opinion, participants that are at high risk for infection (including but not limited to in dwelling catheter, dysphagia with aspiration, decubitus ulcer, history of prior aspiration pneumonia or recurrent severe urinary tract infection)

9. IgG levels below 4.0 g/L, IgA level < 10 mg/dL (IgG and IgA test must be performed no more than 10 days before Day 0
10. Primary immunodeficiency
11. History of malignant neoplasm within the last 5 years
12. History of cervical dysplasia CIN Grade III cervical high-risk human papillomavirus or abnormal cervical cytology other than abnormal squamous cells of undetermined significance (ASCUS) within the past 3 years. The participant will be eligible after the condition has resolved (e.g., follow-up HPV test is negative or cervical abnormality has been effectively treated >1 year ago)
12. Severe, progressive, or uncontrolled renal, hepatic, haematological, gastrointestinal, pulmonary, cardiac, or neurological disease or, in the investigator's opinion, any other concomitant medical condition or significant abnormal laboratory value that places the participant at risk by participating in this trial with the exception of diseases or conditions related to active SLE.
13. Comorbidities not lupus related currently requiring systemic corticosteroid therapy.
14. Evidence of serious suicide risk including any history of suicidal behaviour in the last 6 months and/or any suicidal ideation in the last 2 months or who in the investigator's judgement, pose a significant risk.
15. History of an anaphylactic reaction to parenteral administration of contrast agents, human or murine proteins or monoclonal antibodies.
16. Current drug or alcohol abuse or dependence, or a history of drug or alcohol abuse or dependence within 364 days prior to Day 0.
17. White blood cells (WBC) <1.5 x 10^9/L, Neutrophils <1 x 10^9/L measured up to 10 days before Day 0
18. A history of major organ transplant or haematopoietic stem/cell/marrow transplant or renal transplant.

For peer review only - http://bmjopen.bmj.com/site/about/guidelines.xhtml

Randomisation and Blinding

BEAT Lupus is a double-blind trial. Only the local pharmacist, the unblinded site monitor and the trial statistician will be unblinded to the treatment allocation. All other members of the trial team and patients are blinded to the trial medication throughout the trial. Unblinding will only occur if a participant experiences a serious adverse event for which the clinical management of the SAE will be facilitated by the unblinding of the participant's treatment allocation. All recruited participants will be given a card with contact details for the trial team including emergency contact 24 hours a day, 7 days per week. In the event of unblinding becoming necessary, emergency unblinding can occur at any time via the 24-hour web-based service offered by Sealed Envelope (https://www.sealedenvelope.com/).

After providing written informed consent, participants are randomised to belimumab or placebo treatment using a secure online randomisation service provider, Sealed Envelope. The trial statistician will generate a list of unique BEAT-LUPUS infusion codes which will be sent to Sealed Envelope. The trial pharmacist will dispense the randomised infusion for each participant in accordance with their infusion code. Randomisation will be performed using minimisation, incorporating a random element to ensure balance in the stratifying variables between the two randomised groups, while retaining an element of unpredictability. Stratifying variables are: CD19 count after rituximab at randomisation ($<0.01x10^9/l$ vs $>0.01x10^9/l$) to account for variability in B cell depletion which would affect response; anti dsDNA (positive or negative at first screen); and whether patients have active renal disease at screening.

Trial treatment

Participants either receive intravenous belimumab or placebo 4-8 weeks after the first of two infusions of B cell depletion therapy (one cycle rituximab) administered by trial nurses. The total treatment period (belimumab or placebo) will be 52 weeks (last infusion of belimumab or placebo at week 52) followed by a safety visit at week 56. In the active therapy arm, participants will receive belimumab according to the standard dosage regime. The recommended dosage regimen is 10 mg/kg at 2-week intervals for the first 3 doses and at 4-week intervals thereafter. The participants in the placebo group receive the same volume of normal saline infusions (indistinguishable from active treatment) at the same time points as the active treatment group. The visit schedule is summarised in Figure 1. Trial treatment may be stopped in the event of unacceptable toxicity or adverse event.

For peer review only - http://bmjopen.bmj.com/site/about/guidelines.xhtml

Concomitant medications

Before randomisation, which includes the period when rituximab is administered, treatment is entirely at the physician's discretion, though the rituximab dose is fixed by NHS England (1g administered on two occasions, two weeks apart). All medication is recorded. From the day of randomisation (Day 0, see Figure 1), the maximum dose of mycophenolate is 1g/day with a suggested dose reduction to 500mg/day at 3 months or as soon as possible thereafter. The maximum dose of azathioprine after randomization is 1mg/kg with a suggested dose reduction to 0.5mg/kg by 3 months or as soon as possible thereafter. The maximum dose of methotrexate allowed is 15mg/week with a dose reduction to 10mg/week by 3 months or as soon as possible thereafter. Only one of these three disease modifying drugs (azathioprine, methotrexate and mycophenolate) can be administered post randomisation. No other immunosuppressants (e.g. cyclophosphamide, tacrolimus) are allowed after randomisation except in the case of severe flare. Background anti-malarial drugs are permitted but no new anti-malarials can be started after randomisation. Mepacrine (quinacrine) is acceptable for skin problems resistant to hydroxychloroquine and both can be combined if this regime was started before randomisation. No change in dose of the anti- malarials can be made after randomisation. Participants will be permitted to receive up to 20 mg prednisolone/day from randomisation. It is strongly encouraged that prednisolone should be reduced by 50% by 6 months post randomisation, with a maximum dose of 5mg/day recommended at 6 months if possible. Participants who flare with one BILAG A or two BILAG B scores will be permitted to receive other therapies or increased prednisolone at the discretion of the treating physician. Further cycles of rituximab are not allowed during the trial even in the event of a severe disease flare. Centres are encouraged to follow the British Society for Rheumatology guidelines for the management of systemic lupus erythematosus in adults (14).

Trial endpoints

The primary outcome measure is anti-dsDNA antibody levels at 52 weeks. Anti-dsDNA antibodies often correlate with disease activity (14, 26, 27). This biomarker was also chosen due its correlation with BAFF levels in participants post B cell depletion therapy (19). Serum anti-dsDNA antibodies are analysed using the DIASTAT® anti-dsDNA enzyme-linked immunosorbent assay. All samples are tested in the same central lab at UCL.

Secondary outcome measurements include anti-dsDNA antibody levels at 24 weeks and the proportion of participants with serious adverse events (Box2). The proportion of participants with a moderate (defined as ≥2 new BILAG Bs) or severe disease flare (defined as ≥1 new BILAG A) by 24

For peer review only - http://bmjopen.bmj.com/site/about/guidelines.xhtml

and 52 weeks following randomisation will be assessed, as will the time to a disease flare. Patients who have a severe disease flare will be invited to an additional visit where standard trial data, including serum for the primary endpoint analysis, is collected, particularly if they are withdrawing from trial therapy and may be treated with therapies that are not allowed according to the protocol e.g. rituximab and cyclophosphamide.

Disease activity will be assessed using the British Isles Lupus Assessment Group (BILAG-2004) disease activity index (28) and SLEDAI 2000 (29). C3 and immunoglobulin levels will be recorded. As part of the assessment of response to treatment, we shall calculate the cumulative steroid dose during treatment from randomisation and the proportion of participants decreasing their steroid dose by 50% without flaring from randomisation; or if below 10mg/day at randomisation reducing steroid dose to 5mg/day or who discontinue glucocorticoids with stable disease. Lupus Quality of Life (Lupus QoL) and SF-36 will be used as patient reported outcome measures. C-SSRS (Columbia suicide severity rating scale) will be used to assess suicidality risk.

Data collection and management

All participant data will be collected by members of the clinical trial team as described in the Delegation Log. Data will be recorded on a paper Case Report Forms (CRFs) and entered in to the secure Clinical Trial Database via a secure web interface. All data will be handled in accordance with the Data Protection Act 1998. The participant's initials, date of birth and participant identification number, will be used for identification.

Data entry, coding and security will be the responsibility of a dedicated Data manager based. A custom designed database will be created (using InferMed's MACRO V4) to store all trial data. This will be compliant with all necessary regulatory requirements including an audit trail to record all date/time stamped corrections accompanied by justification/explanation for any data amendments. Monitoring visits will be conducted by the central trial team at all recruiting sites at regular intervals, in accordance with regulatory requirements. The trial may be audited independently by the MHRA.

For peer review only - http://bmjopen.bmj.com/site/about/guidelines.xhtml

BOX 2: OUTCOME MEASURES

Primary outcome measures
Anti-dsDNA-antibody levels at 52 weeks.
Secondary outcome measures
1. Anti-dsDNA antibody levels at 24 weeks
2. Proportion of participants with any adverse events by 52 weeks.
3. Proportion of participants with any serious adverse events by 52 weeks.
4. Proportion of participants with any infections by 52 weeks.
5. Proportion of participants with a severe disease flare (defined as ≥ one BILAG A) by 52 weeks.
6. Proportion of participants with a severe (defined as ≥ one new BILAG A) or a moderate disease flare (defined as ≥ two new BILAG Bs) by 52 weeks.
7. Time to severe disease flare.
8. Proportion of participants with a severe disease flare by 24 weeks.
9. SLEDAI 2000 at 52 weeks.
10. Subject Global Assessment of Disease Activity (SGADA) at 52 weeks.
11. C3 levels at 52 weeks.
12. Immunoglobulin levels at 52 weeks.
13. Cumulative steroid dose from randomisation to 52 weeks.
14. Proportion of participants decreasing steroid dose at randomisation by 50% without experiencing a flare, or if below 10mg/day at randomisation reducing dose to 5mg/day or less at 52 weeks.
15. Lupus Quality of Life (Lupus QoL), SF-36 at 52 weeks
16. C-SSRS (Columbian Suicide Severity Rating Scale) to assess suicidality risk at 52 weeks.

Exploratory outcomes
1. BAFF (measurement of RNA from whole blood) at 52 weeks.
2. Kinetics of B cell repopulation.

Trial sample size calculation

We aim to recruit up to 56 SLE participants (the original sample size of 50 was increased to account for a higher than expected dropout rate) and randomise 28 participants to receive belimumab after rituximab and 28 participants to receive placebo after rituximab. The primary outcome is the estimated difference in anti dsDNA binding antibody levels at 12 months, adjusted for anti dsDNA binding levels at screening. Sample size calculations were performed using Stata Release 13 assuming that anti dsDNA binding antibody levels are log normally distributed. The Standard Deviation of anti dsDNA and the correlation structure assumed were based on two sets of data (a) the study of 35 participants by Carter et al (19) and (b) unpublished data provided by Prof D Isenberg for 67 participants before and 6 months after B cell depletion therapy (Table 1).

For peer review only - http://bmjopen.bmj.com/site/about/guidelines.xhtml

Table 1: Standard deviations and correlations for $\log_e$ anti dsDNA

Dataset	Time point	Log mean	Log sd	Correlation
**Isenberg (n=67)**	**Before rituximab**	6.036	1.393	
	**Follow up**	5.189	1.500	0.612
**Carter (n=35)**	**Before rituximab**	5.091	1.676	
	**Follow up**	5.225	1.781	0.527

On the natural log scale the standard deviation of anti dsDNA at the end of treatment was taken to be 1.7 based on the above data. The correlation between before rituximab and final log anti dsDNA was taken to be 0.55 also based on the above. 22 evaluable participants per group would be able to detect a difference of 1.2 in anti dsDNA binding antibody levels on the natural log scale at the 5% significance level with 80% power. Assuming that 20% of participants fail to attend the 52 week follow-up visit, and taking account of the correlation between screening measurements and 52 week measurements, we aim to recruit 28 participants per group. For example, if we assume that there will be no change in anti dsDNA levels in the belimumab group, then we would be able to detect an increase of 232% in the placebo group as significantly different. To place this increase in context, Carter et al showed that the difference in anti dsDNA binding antibody levels on the natural log scale between those participants who did and did not have a flare was 1.928, corresponding to a 588% increase in anti dsDNA binding levels in participants with a disease flare (19).

Statistical analysis

A CONSORT diagram will be used to describe the course of participants through the trial (Figure 1). Characteristics at screening and randomisation will be summarised by treatment group.

The analysis of the primary outcome will be done using linear regression to estimate the difference in log anti-dsDNA levels between the two groups at 52 weeks, adjusting for the stratifying variables anti-dsDNA (at screening, pre-rituximab), BILAG category (renal, non-renal) (at screening, pre-rituximab), and CD19 count (at randomisation). The analysis will be done on the intention-to-treat principle; all available 52 week measurements will be included regardless of patient's adherence to their randomised treatment. If withdrawals from follow up before week 52 exceed 10%, a supportive analysis of the primary outcome will be done using multiple imputation (with 52-week anti-dsDNA imputed using baseline and any 12, and 24 weeks measurements where available and other auxiliary information) to evaluate the sensitivity of the results to any differential loss to follow

For peer review only - http://bmjopen.bmj.com/site/about/guidelines.xhtml

up. Another supportive sensitivity analysis will be done to estimate the treatment effect for only those patients who adhered to their allocated treatment for 52 weeks. Additionally, if material differences are found between treatment groups in cumulative prednisolone dose from randomisation ($p<0.1$), mediation analyses will be done to evaluate whether the observed effect of belimumab on anti-dsDNA levels is mediated by changes in prednisolone dose.

A similar modelling approach will be adopted for the analysis of other continuous outcomes measured at screening (before rituximab) and at 52 weeks. Fisher's exact test will be used to compare proportions. Two sample t tests or Mann Whitney U tests, depending on the distribution of the data, will be used to evaluate continuous outcomes compared only at the end of the trial. Kaplan-Meier survival curves will be produced for comparisons of time to an event. The statistical analysis will be performed using Stata.

Ethical considerations

The protocol has been reviewed and approved by the Hampstead Research Ethics Committee-London (reference 16/LO/1024) and the MHRA. The trial will be conducted in accordance with ethical principles derived from Good Clinical Practice Guidelines, including the Declaration of Helsinki. Written informed consent (see Supplementary File for consent form) will be collected from all patients by an approved study nurse or physician before the start of any study procedures. Patients will be assigned a participant identification number and all patient information transferred to the sponsor will contain this number, patient initials and date of birth. All Serious Adverse Events (SAE) will be reported to the sponsor within 24 hours. All agreed substantial protocol amendments, will be documented and submitted for ethical and regulatory approval prior to implementation and communication to sites. Protocol version 5 dated 21st January 2019 is the current protocol version and has been approved.

Dissemination

Trial information is available at https://www.isrctn.com/ISRCTN47873003 including study sites all of which are in England, and the results of this trial will be submitted for publication in relevant peer-reviewed publications. Key findings will also be presented at national and international conferences. Published results will be disseminated to investigators at participating sites, who will further disseminate the results to trial participants on request. Authorship of publications will adhere to ICMJE guidelines.

Patient and public involvement

For peer review only - http://bmjopen.bmj.com/site/about/guidelines.xhtml

Feedback gathered from focused patient involvement meetings was used to inform the design of this trial. Patients recognised the need for improving therapies for SLE. Our patient partners suggested that the inclusion and exclusion criteria should not represent a significant barrier to recruitment and that the trial should be as close to "real-world" practise as possible. Two patient advisors are members of the Trial Steering Committee.

Trial Steering Committee

The Trial Steering Committee (TSC) is led by independent chair (Professor Raashid Luqmani) from a non-participating centre, and includes an independent statistician (Professor John Bankart) and one independent clinical expert in SLE (Dr Ceril Rhys-Dillon) as well as the Chief Investigator (Professor Michael Ehrenstein) and members of the trial team, some principal investigators from the participating sites and two patient partners. The TSC provides expert oversight of the trial, agreement of final protocol, reviewing progress of study, recruitment and retention rates, and agreeing changes to the protocol to facilitate the smooth running of the study. The TSC together with the Chief Investigator will oversee the timely analysis and publication of the main trial results and will review requests for data sharing. Full composition of the TSC and terms of reference are available from the corresponding author.

Data Monitoring Committee

An Independent Data Monitoring Committee (IDMC) is chaired by Professor Richard Watts (independent clinical expert) and includes Professor Vern Farewell (independent statistician) and Professor Robert Moots (independent clinical expert) together with the trial statistician. During the period of recruitment to the study, interim analyses of accruing data will be supplied, in strict confidence, to the IDMC. The IDMC will advise the TSC of any issues to safeguard the interests of trial participants including assessment of SAEs and flare rates after 50% of the participants have been recruited and followed up for 6 months to determine whether recruitment should continue (interim safety analysis). All SAEs will be reported in real time to the IDMC with a clinical narrative, including an assessment of attribution to Belimumab. Full composition of the IDMC and terms of reference are available from UCL CCTU.

Discussion

For peer review only - http://bmjopen.bmj.com/site/about/guidelines.xhtml

This clinical trial was developed through investigation and analysis of clinical and laboratory data of lupus patients receiving rituximab. In particular, we noted increased serum BAFF levels correlated with a rise in anti-DNA antibodies at the time of flare in lupus patients post rituximab (19). We therefore hypothesised that lupus patients treated with belimumab following rituximab therapy will have lower anti-dsDNA antibody levels compared to those that have received rituximab alone, and that this combination would have greater efficacy than rituximab alone. Given the paucity of novel therapies, and the many clinical trials that have failed to show efficacy for SLE, a combination of biologic therapies with complementary effects given in succession may be required to control disease. If the results of this trial are promising, a larger trial will be required of sufficient power to detect improved clinical outcomes. Indeed, a larger trial is already underway (BLISS BELIEVE NCT03312907) testing whether rituximab given after belimumab confers an additional benefit compared to belimumab alone (30). The order of administration of these two biologics in BLISS BELIEVE is reversed compared to BEAT-LUPUS, and there is no rituximab alone arm. The administration of rituximab first allows for the detection of adverse events that occur in the 4-8 week period after rituximab and before randomisation. A second screen 7-10 days before randomisation will exclude patients based on these adverse events. CALIBRATE (NCT02260934), a clinical trial for lupus nephritis uses a similar sequence of these two biologics to the trial reported here, though cyclophosphamide is administered with the rituximab, and higher doses of concomitant medications are allowed.

Recruitment to lupus trials has been challenging in the UK, and few academic led interventional SLE trials in the UK have met their recruitment target. The last academic led lupus trial in the UK not subject to early termination because of poor recruitment commenced in 1997 and completed recruitment in 2005 (though even this open label trial's recruitment target had to be reduced due to slow enrolment) (31). We endeavoured to optimise recruitment by designing a protocol that would be as close to real life clinical practice as possible. Consequently, there are no therapeutic restrictions before patients are randomised (to receive either belimumab or placebo) giving freedom to the treating physician including the possibility of administering high dose corticosteroids in conjunction with rituximab. Rituximab had to be administered 4-8 weeks before randomisation and is administered in accordance with the NHS England's Interim Commissioning Policy (NHS ENGLAND A13/PS/a) for patients with refractory lupus (14). This policy only allows rituximab to be given to patients who have failed to respond adequately to either mycophenolate or cyclophosphamide, unless contraindicated, or requiring unacceptably high doses of corticosteroids. Thus, the results of this trial will be relevant to lupus patients that are refractory to or intolerant of conventional therapy, the patient group for which this combination therapy is most likely to be funded. The study

For peer review only - http://bmjopen.bmj.com/site/about/guidelines.xhtml

provides information about rates of recruitment to lupus trials in England which could inform the development and feasibility of other UK based trials planned in the future. Recruitment was aided through NHS England's policy applicable to biological therapies for SLE which encourages physicians and their patients to consider interventional clinical trials (NHS ENGLAND A13/PS/a) (14).

The trial protocol offers physicians some flexibility with the use of concomitant immunosuppressant and oral prednisolone after randomisation. However, because of safety concerns, and to maximise the chances of revealing any potential difference between the two treatment arms, corticosteroids are limited to a maximum dose of 20mg/day at randomisation and it is strongly encouraged to taper the dose by at least 50% by 6 months. Moreover, immunosuppressant doses are also restricted. It can be difficult to achieve reductions in both corticosteroids and immunosuppressants in patients who, by the nature of the inclusion criteria, are more resistant to therapy. Our strategy is to advise that reduction in corticosteroid dose should be prioritised over tapering immunosuppressants due to the former's more problematic side effects, and its ability to mask the difference between treatment arms in lupus trials.

This trial has some limitations. Firstly, the sample size is relatively small because at the time of the trial's inception there were no published safety data on the combination of rituximab and belimumab and an earlier phase, smaller trial was advised. Therefore, the trial is powered on anti-DNA antibodies rather than a clinical endpoint. To limit the risk of adverse events due to immunosuppression, the dose of concomitant DMARDs is lower than routinely prescribed for patients with active disease, particularly renal patients receiving mycophenolate. This may reduce recruitment of patients with active nephritis by some physicians, although this restriction in dosage could also be an advantage with respect to distinguishing between active drug and placebo. The serological inclusion criteria enable patients with a confirmed positive dsDNA antibody within the last 5 years to participate in the trial rather than patients who have raised anti-DNA antibodies at screen. This approach was taken to maximise recruitment but also with the recognition that flares post rituximab can, in a proportion of patients, be associated with increased anti-DNA antibody levels above that observed when rituximab is first commenced (15).

The development of this trial has arisen directly from analysis of the immunologic changes following B cell depletion therapy. Building on this experimental medicine approach, analysis of B cell phenotype has been incorporated into this trial to understand how sequential targeting of B cells and BAFF affect B cell numbers and phenotype. If the overall results from this trial are encouraging, a larger definitive trial using time to flare as the primary outcome may be considered after taking

For peer review only - http://bmjopen.bmj.com/site/about/guidelines.xhtml

into account the results from other similar trials currently underway. The increasing interest in this combination therapy has the potential to offer a novel treatment option for patients with active lupus.

For peer review only

For peer review only - http://bmjopen.bmj.com/site/about/guidelines.xhtml

Funding

This trial is supported by Versus Arthritis (grant number 20873) and the UCLH Biomedical Research Centre (BRC). GSK are providing belimumab free of charge, as well as additional funding. The MRC (MASTERPLANS CONSORTIUM) is supporting some of the experimental medicine applied to samples from this trial.

GSK had no role in the design of this study and will not have any role during its execution, analyses, interpretation of the data, or decision to submit results. Versus Arthritis and the MRC reviewed the relevant grant proposals and monitor progress of relevant aspects of the study but will not play any role in the analyses, interpretation of data, or decision to submit results.

Conflict of interest statement

MRE has received grant/research support from GSK. CG has been a member of the speakers bureau for GSK and has received consultancy fees for attending advisory boards.

Contributorship

MRE conceived of the study. MRE, CG, CD and DAI initiated the study design. PM and KC and CD provided statistical expertise in clinical trial in clinical trial design. AJ, MRE, CG, CD, FI and EC assisted with protocol development. AJ, MRE, PM, CD wrote the manuscript, all authors reviewed the manuscript. MRE, DAI, CG and CD are grant holders.

Acknowledgements

We acknowledge the important contribution of the BEAT Lupus Trial Steering Committee, the Data Monitoring Committee, and all the patients participating in the trial or involved in its development. All authors reviewed and edited the draft version of the manuscript and approved the final version submitted. We are indebted to the BEAT-LUPUS Trial Collaborators at the recruiting sites, the British Isles Lupus Assessment Group (BILAG) and The NIHR Musculoskeletal Translational Research Collaboration for their constant advice and support. The trial is sponsored by University College London, Gower Street

London, WC1E 6BT, United Kingdom, +44 20 7679 6163, ctu.beatlupus@ucl.ac.uk.

Trial registration number: ISRCTN47873

https://www.isrctn.com/ISRCTN47873003

Date assigned to the registry: 28/11/2016

For peer review only - http://bmjopen.bmj.com/site/about/guidelines.xhtml

Figure 1 Schedule of Trial Visits

REFERENCES

1. Isenberg DA, Merrill JT. Why, why, why de-lupus (does so badly in clinical trials). Expert Rev Clin Immunol. 2016;12(2):95-8.
2. Galarza-Maldonado C, Kourilovitch MR, Molineros JE, Cardiel MH, Zurita L, Soroka NF, et al. The administration of low doses of rituximab followed by hydroxychloroquine, prednisone and low doses of mycophenolate mofetil is an effective therapy in Latin American patients with active systemic lupus erythematosus. Autoimmunity reviews. 2010;10(2):108-11.
3. Leandro MJ, Edwards JC, Cambridge G, Ehrenstein MR, Isenberg DA. An open study of B lymphocyte depletion in systemic lupus erythematosus. Arthritis & Rheumatism. 2002;46(10):2673-7.
4. Vital EM, Dass S, Buch MH, Henshaw K, Pease CT, Martin MF, et al. B cell biomarkers of rituximab responses in systemic lupus erythematosus. Arthritis & Rheumatism. 2011;63(10):3038-47.
5. Aguiar R, Araújo C, Martins-Coelho G, Isenberg D. Use of Rituximab in Systemic Lupus Erythematosus: A Single Center Experience Over 14 Years. Arthritis Care & Research. 2017;69(2):257-62.
6. Ramos-Casals M, Brito-Zerón P, Muñoz S, Soto M-J. A systematic review of the off-label use of biological therapies in systemic autoimmune diseases. Medicine. 2008;87(6):345-64.
7. Díaz-Lagares C, Croca S, Sangle S, Vital EM, Catapano F, Martínez-Berriotxoa A, et al. Efficacy of rituximab in 164 patients with biopsy-proven lupus nephritis: pooled data from European cohorts. Autoimmunity reviews. 2012;11(5):357-64.
8. Witt M, Grunke M, Proft F, Baeuerle M, Aringer M, Burmester G, et al. Clinical outcomes and safety of rituximab treatment for patients with systemic lupus erythematosus (SLE)–results from a nationwide cohort in Germany (GRAID). Lupus. 2013;22(11):1142-9.
9. McCarthy EM, Sutton E, Nesbit S, White J, Parker B, Jayne D, et al. Short-term efficacy and safety of rituximab therapy in refractory systemic lupus erythematosus: results from the British Isles Lupus Assessment Group Biologics Register. Rheumatology. 2017;57(3):470-9.
10. Merrill J, Buyon J, Furie R, Latinis K, Gordon C, Hsieh H, et al. Assessment of flares in lupus patients enrolled in a phase II/III study of rituximab (EXPLORER). Lupus. 2011;20(7):709-16.
11. Merrill JT, Neuwelt CM, Wallace DJ, Shanahan JC, Latinis KM, Oates JC, et al. Efficacy and safety of rituximab in moderately-to-severely active systemic lupus erythematosus: the randomized, double-blind, phase II/III systemic lupus erythematosus evaluation of rituximab trial. Arthritis & Rheumatism: Official Journal of the American College of Rheumatology. 2010;62(1):222-33.
12. Rovin BH, Furie R, Latinis K, Looney RJ, Fervenza FC, Sanchez-Guerrero J, et al. Efficacy and safety of rituximab in patients with active proliferative lupus nephritis: the Lupus Nephritis Assessment with Rituximab study. Arthritis & Rheumatism. 2012;64(4):1215-26.
13. Fanouriakis A, Kostopoulou M, Alunno A, Aringer M, Bajema I, Boletis JN, et al. 2019 update of the EULAR recommendations for the management of systemic lupus erythematosus. Annals of the Rheumatic Diseases. 2019;78(6):736-45.

For peer review only

14. Gordon C, Amissah-Arthur M-B, Gayed M, Brown S, Bruce IN, D'Cruz D, et al. The British Society for Rheumatology guideline for the management of systemic lupus erythematosus in adults. Rheumatology. 2017;57(1):e1-e45.
15. Lazarus MN, Turner-Stokes T, Chavele K-M, Isenberg DA, Ehrenstein MR. B-cell numbers and phenotype at clinical relapse following rituximab therapy differ in SLE patients according to anti-dsDNA antibody levels. Rheumatology. 2012;51(7):1208-15.
16. Iaccarino L, Bartoloni E, Carli L, Ceccarelli F, Conti F, De SV, et al. Efficacy and safety of off-label use of rituximab in refractory lupus: data from the Italian Multicentre Registry. Clinical and experimental rheumatology. 2015;33(4):449-56.
17. Cambridge G, Isenberg DA, Edwards J, Leandro MJ, Migone T-S, Teodorescu M, et al. B cell depletion therapy in systemic lupus erythaematosus: relationships among serum B lymphocyte stimulator levels, autoantibody profile and clinical response. Annals of the rheumatic diseases. 2008;67(7):1011-6.
18. Vallerskog T, Heimbürger M, Gunnarsson I, Zhou W, Wahren-Herlenius M, Trollmo C, et al. Differential effects on BAFF and APRIL levels in rituximab-treated patients with systemic lupus erythematosus and rheumatoid arthritis. Arthritis research & therapy. 2006;8(6):R167.
19. Carter LM, Isenberg DA, Ehrenstein MR. Elevated Serum BAFF Levels Are Associated With Rising Anti–Double-Stranded DNA Antibody Levels and Disease Flare Following B Cell Depletion Therapy in Systemic Lupus Erythematosus. Arthritis & Rheumatism. 2013;65(10):2672-9.
20. Ehrenstein MR, Wing C. The BAFFling effects of rituximab in lupus: danger ahead? Nature Reviews Rheumatology. 2016;12(6):367.
21. Cheema GS, Roschke V, Hilbert DM, Stohl W. Elevated serum B lymphocyte stimulator levels in patients with systemic immune–based rheumatic diseases. Arthritis & Rheumatism: Official Journal of the American College of Rheumatology. 2001;44(6):1313-9.
22. Stohl W, Metyas S, Tan SM, Cheema GS, Oamar B, Xu D, et al. B lymphocyte stimulator overexpression in patients with systemic lupus erythematosus: longitudinal observations. Arthritis & Rheumatism: Official Journal of the American College of Rheumatology. 2003;48(12):3475-86.
23. Petri M, Stohl W, Chatham W, McCune WJ, Chevrier M, Ryel J, et al. Association of plasma B lymphocyte stimulator levels and disease activity in systemic lupus erythematosus. Arthritis & Rheumatism: Official Journal of the American College of Rheumatology. 2008;58(8):2453-9.
24. Navarra SV, Guzmán RM, Gallacher AE, Hall S, Levy RA, Jimenez RE, et al. Efficacy and safety of belimumab in patients with active systemic lupus erythematosus: a randomised, placebo-controlled, phase 3 trial. The Lancet. 2011;377(9767):721-31.
25. Furie R, Petri M, Zamani O, Cervera R, Wallace DJ, Tegzová D, et al. A phase III, randomized, placebo-controlled study of belimumab, a monoclonal antibody that inhibits B lymphocyte stimulator, in patients with systemic lupus erythematosus. Arthritis & Rheumatism. 2011;63(12):3918-30.
26. Isenberg D, Manson J, Ehrenstein M, Rahman A. Fifty years of anti-ds DNA antibodies: are we approaching journey's end? 2007.
27. Floris A, Piga M, Cauli A, Mathieu A. Predictors of flares in Systemic Lupus Erythematosus: Preventive therapeutic intervention based on serial anti-dsDNA antibodies assessment. Analysis of a monocentric cohort and literature review. Autoimmunity Reviews. 2016;15(7):656-63.

For peer review only

28. Isenberg DA, Rahman A, Allen E, Farewell V, Akil M, Bruce IN, et al. BILAG 2004. Development and initial validation of an updated version of the British Isles Lupus Assessment Group's disease activity index for patients with systemic lupus erythematosus. Rheumatology (Oxford). 2005;44(7):902-6.
29. Gladman DD, Ibanez D, Urowitz MB. Systemic lupus erythematosus disease activity index 2000. The Journal of rheumatology. 2002;29(2):288-91.
30. Teng YKO, Bruce IN, Diamond B, Furie RA, van Vollenhoven RF, Gordon D, et al. Phase III, multicentre, randomised, double-blind, placebo-controlled, 104-week study of subcutaneous belimumab administered in combination with rituximab in adults with systemic lupus erythematosus (SLE): BLISS-BELIEVE study protocol. BMJ Open. 2019;9(3):e025687.
31. Griffiths B, Emery P, Ryan V, Isenberg D, Akil M, Thompson R, et al. The BILAG multi-centre open randomized controlled trial comparing ciclosporin vs azathioprine in patients with severe SLE. Rheumatology. 2010;49(4):723-32.

For peer review only

Figure 1 Schedule of Trial visits

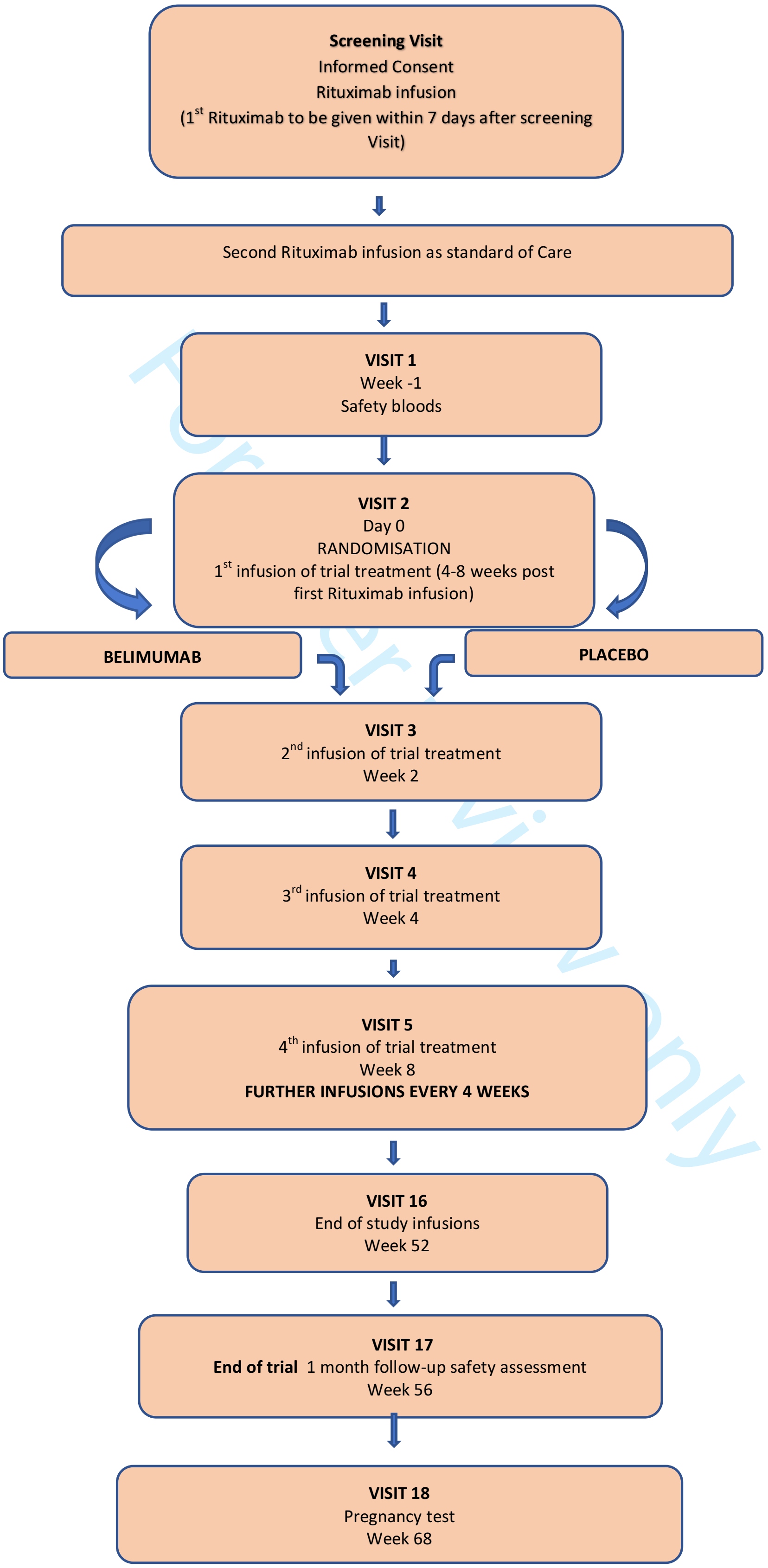

For peer review only

Comprehensive Clinical Trials Unit

[to be printed on hospital headed paper]

Patient Information Sheet and informed consent form

Study title: BEAT LUPUS

Safety and efficacy of BElimumab After B cell depleTion therapy in systemic LUPUS erythematosus

Protocol Reference Number: CTU/2013/096

We would like to invite you to take part in our clinical study. University College London (UCL) is responsible for the study. Taking on this responsibility is called being the study sponsor. Before you decide whether or not to take part, we would like you to understand why the study is being done and what it would involve for you. One of our team will go through the information sheet with you and answer any questions you have. We‘d suggest this should take about 20 minutes.
Ask us if there is anything that is not clear. Talk to others about the study if you wish. Take time to decide whether or not you wish to take part.

1. What is the purpose of the study?

This study aims to find out whether a drug called Belimumab (Benlysta) when used after B cell depletion therapy is both safe and effective in reducing systemic lupus erythematosus (lupus) disease activity. B cell depletion therapy (normally rituximab) is being given as part of your normal treatment because your lupus is active. B cell depletion therapy removes a type of immune cell called B cells from the body. B cells can cause disease in lupus patients. Although patients respond to B cell depletion therapy the disease can quickly return. This is likely to be because a chemical (also known as a stimulating factor) called BAFF increases in the body once treatment ends, and switches the lupus back on. Belimumab is a drug that stops BAFF from working and has been shown to work in patients with lupus. We will treat you with belimumab soon after B cell depletion therapy and we hope this combined treatment approach will prevent the lupus from coming back after rituximab therapy. However, we don't know yet whether giving belimumab is any better than simply having B cell depletion therapy alone.

2. Why have I been invited?

You have been invited because you have lupus and your disease has not responded to treatment with medication such as steroids and mycophenolate. Patients whose disease does not normally respond to these treatments can receive rituximab as part of their normal care. We aim to recruit 50 lupus patients to this study who have received B cell depletion therapy in the last month or so.

3. Do I have to take part?

It is up to you to decide to join the study. We will describe the study and go through this information sheet with you. If you agree to take part, we will then ask you to sign a consent form. You are free to withdraw your consent at any time without giving a reason. This will not affect the standard of care you receive.

For peer review only - http://bmjopen.bmj.com/site/about/guidelines.xhtml

4. What will happen to me if I take part?

If you wish to take part, you will be asked a series of questions to see if you might be eligible to join the study. If you are eligible at this point you will be asked to attend the hospital to be asked more details about your condition and be examined and have blood and urine tests.

If this assessment confirms you are eligible, you will be entered into the study and will be invited to attend the hospital again.

You will return to hospital to have further blood tests, and an ECG (a test of your heart). If you are a woman and of child bearing age then you will have a pregnancy test at each visit and as noted below you will be asked to use appropriate contraception so that you will not get pregnant whilst you are in the study and within 16 weeks after the last dose of study treatment. Tests will be run for serious infections such as HIV or Hepatitis. All these tests are tests you would have as part of your normal care.

This is a randomized study. We don't know the best way of treating patients after B cell depletion therapy. To find out, we need to compare a new treatment (belimumab) with usual treatment. We will put people into groups and give one group belimumab and the other group the usual treatment. The results are compared to see if one is better. To try to make sure the groups are the same to start with, each patient is put into a group by chance (randomly). The decision as to which group you will be put in will be made by a computer.

Half the patients receive belimumab and half receive placebo. Both treatments will be given by intravenous infusion. The placebo treatment will look the same as belimumab but contains no active drug. The reason for including a placebo is so neither you nor your doctor knows whether belimumab has been given. Sometimes simply knowing what treatment has been given can affect the way a patient is feeling. We will be able to compare the information from patients in the belimumab group, with the information from the placebo group and be able to determine what effect belimumab is having on Lupus.

Your doctor will be able to find out which group you are in should there be a need to, but this does not usually happen while you are taking part in the study.

All patients will be invited to attend for an infusion of the study medication which will last an hour (but you may be monitored for a few hours after the first couple of infusions) and you will repeat treatments and assessments, usually every four weeks.

The results of the study will be available for general use by researchers in the field of Lupus, to ensure that the information gathered from the study is put to maximum use. Your personal details will not be revealed at any stage.

5. Expenses and payments

We will pay your travel expenses as a result of taking part in the study.

6. What will I have to do?

If you join this study, you will need to come to the hospital 17 times over a period of 13 months as well as the visit when you decide whether you want to join the study.

We will ask you not to participate in studies of other therapies during the course of this study. There are no other restrictions on your lifestyle by being part of this study.

For peer review only - http://bmjopen.bmj.com/site/about/guidelines.xhtml

Your doctor can give you other treatments during the study such as prednisolone and other immunosuppressants with your agreement. Some of these treatments may be slowly reduced. You may need to remain on small doses of prednisolone and immunosuppressants until the end of the study. If your disease worsens during the study your doctor can increase these standard treatments with your agreement.

Trial Summary Assessments
Screening:

- Study discussion and patient consent
- Physical examination, review of medication, and blood tests
- Questionnaire completion
- Urine check
- Pregnancy check
- Research blood tests. These samples will be stored using a code and maybe sent to research labs outside of the hospital. All data are kept confidential.

Visit 1

- Physical examination, review of medication, and blood tests
- Questionnaire completion
- Urine check
- Pregnancy check
- ECG

Visit 2

- Physical examination, review of medication, and blood tests
- Questionnaire completion
- Urine check
- Pregnancy check
- Research blood tests. These samples will be stored using a code and maybe sent to research labs outside of the hospital. All data are kept confidential
- **Randomisation to Belimumab or placebo infusions**

Visits 3-16
Are at Week 2, 4, 8, 12, 16, 20, 24, 28, 32, 36, 40, 44, 48 and 52 weeks after randomisation

- Physical examination, review of medication, and blood tests
- Questionnaire completion
- Urine check
- Pregnancy check
- ECG (Visit 10,16)
- Research blood tests. These samples will be stored using a code and maybe sent to research labs outside the hospital. All data are kept confidential. The amount of blood taken will vary at each visit but will not be more than 120ml (about 6 tablespoons).
- Belimumab or placebo depending on which group you are in together with your usual treatments.

Follow up, Visit 17
Week 56

- Physical examination, review of medication, and blood tests
- Questionnaire completion
- Urine check
- Pregnancy check
- Research blood tests. These samples will be stored using a code and maybe sent to research labs outside the hospital. All data are kept confidential.

For peer review only - http://bmjopen.bmj.com/site/about/guidelines.xhtml

Visits 18
Week 68

- Pregnancy check
- Follow up phone call (male participants only)

Extra or unplanned visits
You may be asked to come for extra visits if your doctor believes your disease may be getting worse. If this happens you may be asked to have some of the tests that are usually performed at a study visit.

7. What are the alternatives for diagnosis or treatment?
The rituximab treatment can be repeated without belimumab. Prednisolone and other immunosuppressants can be used to treat your lupus.

8. What are the possible benefits of taking part?
During the study you will have a regular review of your Lupus by an experienced Lupus specialist. Two large studies each involving just over 800 patients with lupus have demonstrated that belimumab is an effective treatment for lupus resulting in fewer flares in disease and allows patients to reduce their steroid dose. It has not been studied in patients with severe lupus kidney disease or severe lupus disease affecting the brain. Other smaller studies suggest that belimumab after rituximab may be helpful to relieve some lupus symptoms and prevent lupus flares. We cannot promise the study will help you but the information we get from this study will help improve the treatment of lupus.

9. What are the possible disadvantages and risks of taking part?

The main risk of taking part is that belimumab has not been given after drugs like rituximab in a research study like this one before and we don't know if this is a safe combination.

Another disadvantage of taking part in this study is the need to spend approximately an hour reclining whilst having an infusion in to a vein in your arm on 15 occasions. If you feel that this would cause you too much discomfort, then you should not take part.

You will need to have blood tests at each assessment which carries the risk of bruising, discomfort or fainting.

10. What are the side effects of belimumab given soon after rituximab?
You should have been given appropriate information about your current treatment and if this has not happened please contact your local research team, they will be able to discuss details of potential side effects that might be associated with the treatment administered in BEAT Lupus.

Belimumab has been studied in patients with lupus but not after treatment with rituximab. Details of the possible side effects given below are taken from the results of two large trials of belimumab in lupus mentioned in part 8 of this leaflet. In those two trials belimumab caused few side effects and the number of infections was not increased compared to patients who received placebo. Patients given both rituximab followed by belimumab may experience worse or fewer side effects than either drug alone and some effects we may not be able to predict.

Serious Side Effects: Some patients receiving belimumab have experienced serious side effects. These have included:

For peer review only - http://bmjopen.bmj.com/site/about/guidelines.xhtml

Risk of Serious Allergic reactions:

The most common side effects that happen during or soon after a belimumab dose are nausea, diarrhoea, and fever. These side effects are usually not severe.

Some patients (up to 1 out of 100) have more severe side effects. These may happen because of an allergic reaction. Patients who have had allergic reactions before may be more likely to have allergic reactions to belimumab. These reactions happen most often on the day of or the day after the first or second belimumab dose. They can be very serious but rarely cause death. With these reactions there may be swelling of the face, lips, mouth, or tongue; wheezing, difficulty in breathing or shortness of breath; rash or itchy raised bumps (welts or hives), slow heartbeat, high or low blood pressure, or dizziness. Get medical help right away if you get any of these problems during the study.

Your study doctor or study nurse will watch you closely during and after each infusion for signs of a reaction. You will need to stay in the clinic for 3 hours after the first and second infusions. Your doctor may decide to watch you for more than 3 hours after a treatment or after more than 2 treatments. Sometimes allergy medicines or medicines like paracetamol are given before an infusion. It is not known if this stops you from having allergic side effects, or if it will make allergic side effects less severe.

Sometimes allergic side effects can occur after you have left the hospital. These types of reactions generally occur 5-10 days after a dose of medication (but can occur before or after that time) and may include a combination of symptoms such as rash, nausea, fatigue, muscle aches, headache, and/or facial swelling. If you experience these symptoms, particularly if you experience a combination of such symptoms, contact your study doctor or study nurse.

Risk of Infection

Because of the type of medicine it is, belimumab may make it more likely for you to get an infection. Tell your doctor if you get chills or a fever or any other sign that makes you think you may have an infection. If you have an infection that keeps coming back or is hard to get rid of, you should not be treated with belimumab. Make sure you talk to your study doctor if you have or get these kinds of infections.

Progressive multifocal leukoencephalopathy (PML) is a serious and life threatening brain condition. Your chance of getting PML may be higher if you are treated with medicines that weaken your immune system, including belimumab. Tell your doctor immediately if you have memory loss, trouble thinking, difficulty with talking or walking, loss of vision, or similar problems

Risk of Cancer

So far, in the many thousands of patients who have received belimumab the risk of cancer has not increased compared to patients who do not get belimumab. However, we cannot say for definite that belimumab does not increase the risk of cancer to a small degree.

Vaccines

Vaccines help your body fight infections. If you have a vaccine while you are taking belimumab, the vaccine might not work as well as if you weren't taking belimumab. Also, some types of vaccines may not be safe for you to have while you are taking belimumab. Make sure you check with your study doctor before having any vaccine.

For peer review only - http://bmjopen.bmj.com/site/about/guidelines.xhtml

Male fertility

Your doctor will explain that the effect on human male fertility is unknown, therefore if you are a male patient entering this trial, you must use a form of effective contraception throughout the trial and for at least 4 months after the last dose of study medicine. Your study doctor or nurse will phone you 16 weeks after your last trial treatment to obtain information as to whether there has been any reported pregnancies.

Pregnancy and Contraception

You should not take part in this study if you are pregnant. Mothers should not breastfeed a baby while on this study.

You should not get pregnant while you are in this study. If you are a woman who is able to get pregnant you must agree to use a type of birth control that works well. Your doctor will explain to you that the effects of the study treatment on pregnancy and the developing foetus are not yet known or are not fully understood at this time, therefore you will have to use one of these types of birth control while you are in this study and for at least 4 months after the last dose of study medicine. Your study doctor or study nurse will tell you about the choices of birth control that you can use in this study. Even if you are using birth control, you will have pregnancy testing at every visit during the study and up to 4 months after your last infusion of the study drug. Tell your study doctor or study nurse if you become pregnant during the study or within 4 months after the last infusion of the study drug...

If you have lupus and are taking a drug for your lupus called mycophenolate (for example CellCept®), additional methods of birth control will be required unless you avoid sexual intercourse completely (abstinence as defined above). Discuss required methods of contraception with your study doctor. Birth control pills may not work as well when you are taking mycophenolate and you could become pregnant.

Serious Mental Health Problems and Suicide:

Mental health problems, such as depression, suicide, trouble sleeping and anxiety are common in patients with lupus and were reported in patients receiving belimumab. Depression, thoughts of suicide and attempting to commit suicide have been reported more frequently in patients receiving belimumab than in patients who did not receive belimumab. Committing suicide has also been reported in patients who took belimumab.

Tell your study doctor or study nurse right away or go to a hospital right away if you attempt to commit suicide, action dangerous impulses, or have thoughts of suicide, hurting yourself, or dying. Also tell your doctor if you have been depressed, worried or anxious.. Tell your doctor even if these problems are not bothering you a lot right now. Also tell your study doctor if you are having any mood problems, are not acting or feeling like yourself, or are having behaviour problems. If your family or friends have told you that they think you have these problems, tell your doctor, even if you don't agree.

You will be asked questions about your mood and any thoughts of harming yourself that you may have had prior to or during the study.

You will be asked to complete a test administered by your study doctor or study nurse to evaluate your risk for suicide. Mental health problems such as depression, suicide, trouble sleeping, and anxiety are common in patients with lupus and were reported in patients receiving belimumab. Depression, thoughts of suicide and attempting to commit suicide have been reported more frequently in patients receiving belimumab than in patients who did not receive belimumab.

For peer review only - http://bmjopen.bmj.com/site/about/guidelines.xhtml

Committing suicide has also been reported in patients who took belimumab. You will be asked questions about your mood and any thoughts of self-harm that you may have. Tell your study doctor if you have new or worse depression or anxiety, thoughts about suicide or dying or other unusual changes in behaviour or mood during the study.

11. Combination of belimumab and rituximab

There is no available information from previous or ongoing clinical studies regarding the side effects of administering belimumab and rituximab in combination. Therefore, there is the possibility that the combination of these two medicines may increase the side effects potentially caused by either medicine alone or may cause new side effects that are not known now.

Both belimumab and rituximab may increase your risk of infection and/or suffering allergic side effects when the drug is given to you. It is possible that this risk may be increased when belimumab and rituximab are administered close together. Your doctor can explain to you the steps that will be taken to reduce this risk.

In addition, it is also possible that the risks of cancer and effects on your mood (feeling anxious or depressed) could be increased when both belimumab and rituximab are administered close together.

12. What happens when the research study stops?

Belimumab treatment will not be automatically provided to any patient participating in this study after its completion. All patients will continue to receive their regular lupus medication and follow up appointments with their referring doctor. Please note that patients will also remain under the study team for 30 days after stopping belimumab or placebo. It is also possible that you may receive more rituximab after the study as part of normal care or belimumab if these are available under the NHS. As belimumab is relatively expensive its use is currently restricted by the NHS, and it is not possible to predict whether an individual patient would be eligible to receive belimumab. The results of this study may help to make belimumab available after rituximab but only if the results show that belimumab after rituximab helps patients with lupus. As this is a small trial further studies may be needed.

It is possible that we may stop the study early because the results look like the belimumab is not working, or is unsafe. If this happens the reasons will be discussed with you and your doctor will talk to you about your treatment options.

13. What if there is a problem?

Every care will be taken in the course of this clinical study. However, in the unlikely event that you are injured by taking part, compensation may be available.

If you suspect that the injury is result of UCL's or your hospital's negligence then you may be able to claim compensation. After discussing with your clinical study doctor, you should make the claim in writing to your study doctor who will then pass the claim to the Sponsor's Insurers, via the Sponsor's office. You may have to bear the costs of the action initially, and you should consult a lawyer about this. Participants may also be able to claim compensation for injury caused by participation in this clinical study without the need to prove negligence on the part of University College London or another party. All claims should be made in writing to Professor Ehrenstein.

Regardless of this, if you wish to complain, or have any concerns about any aspect of the way you have been approached or treated by members of staff or about any side effects (adverse events) you may have experienced due to your participation in the study, the normal National Health Service complaints mechanisms are available to you. Please ask your study doctor if you would like more

For peer review only - http://bmjopen.bmj.com/site/about/guidelines.xhtml

information about this. Details can also be obtained from the Department of Health website: http://www.dh.gov.uk. Alternatively local contact details for complaints:

Name *[insert name]* **Tel. Number: *[insert number]***

14. What will happen to information about me collected during the trial?

University College London is the sponsor for this study based in the United Kingdom. We will be using information from you and/or your medical records in order to undertake this study and will act as the data controller for this study. This means that we are responsible for looking after your information and using it properly. University College London will keep identifiable information about you for a minimum of 10 years after the study has finished.

Your rights to access, change or move your information are limited, as we need to manage your information in specific ways in order for the research to be reliable and accurate. If you withdraw from the study, we will keep the information about you that we have already obtained. To safeguard your rights, we will use the minimum personally-identifiable information possible.

You can find out more about how we use your information at www.ucl.ac.uk/cctu/use-of-data

[insert name of NHS site] will collect information from you and/or your medical records for this research study in accordance with our instructions.

[insert name of NHS site] will keep your name, and contact details confidential and will not pass this information to University College London. ***[insert name of NHS site]*** will use this information as needed, to contact you about the research study, and make sure that relevant information about the study is recorded for your care, and to oversee the quality of the study. Certain individuals from University College London and regulatory organisations may look at your medical and research records to check the accuracy of the research study. University College London will only receive information without any identifying information. The people who analyse the information will not be able to identify you and will not be able to find out your name, or contact details.

[insert name of NHS site] will keep identifiable information about you from this study for 10 years after the study has finished.

When you agree to take part in a research study, the information about your health and care may be provided to researchers running other research studies in this organisation and in other organisations. These organisations may be universities, NHS organisations or companies involved in health and care research in this country or abroad. Your information will only be used by organisations and researchers to conduct research in accordance with the UK Policy Framework for Health and Social Care Research.

This information will not identify you and will not be combined with other information in a way that could identify you. The information will only be used for the purpose of health and care research, and cannot be used to contact you or to affect your care. It will not be used to make decisions about future services available to you, such as insurance.

15. What if relevant new information becomes available?

Sometimes we get new information about the treatment being studied. If this happens, we will tell you about it and discuss whether you want to or should continue in the study. If you decide not to carry on, we will make arrangements for your care to continue. If you decide to continue in the study we will ask you to sign an updated consent form.

For peer review only - http://bmjopen.bmj.com/site/about/guidelines.xhtml

16. What will happen if I don't want to carry on with the study?

You may withdraw from the study at any time without giving a reason. However, if a patient withdraws from a clinical study it can affect how the results are analysed, so we will ask you to think carefully about participating in the study and attending for all visits before you agree to take part. Information collected during the study may still be used. We will ask to continue to see you to monitor your progress but this is not compulsory. If you do not wish to continue follow up, we will request to see you for an exit check-up. Any stored blood samples that can still be identified as yours will be destroyed if you wish.

17. Will my GP be informed of my involvement?

With your consent, your GP, and other doctors who may be treating you, will be notified that you are taking part in this study. If there are any changes in your health requirements as a result of taking part in the study, your GP will again be informed. You can only join this study if you agree to your GP being informed.

18. What will happen to any samples I give?

Blood samples and urine samples will be collected at every study visit. Some of these samples will be tested at other research laboratories and then securely stored in case any need arises to test them as part of future research. These samples will be accessible only to the staff of the hospital laboratories. Those samples that are sent to research laboratories will be coded and there will be no patient identifiable information on these tubes. They will not be transferred out of the UK.

19. Will any genetic tests be done?

The tests on the research bloods may include genetic testing.

20. What will happen to the results of the research study?

The results of the study will be available after it finishes and will usually be published in a medical journal or presented at a scientific conference. The data will be anonymous and none of the patients involved in the study will be identified in any report or publication. Should you wish to see the results, or the publication, please ask your study doctor.

21. Who is organising and funding the research?

This research is being funded by Arthritis Research UK, GSK and UCLH Biomedical Research Centre, and the drug is being provided free of charge by GSK. The study is being co-ordinated by University College London Comprehensive Clinical Trials Unit. The doctors looking after you are not being paid for including you and have no conflicts of interest.

22. Who has reviewed the study?

All research in the NHS is looked at by an independent group of people, called a Research Ethics Committee, to protect your interests. This study has been reviewed and given favourable opinion by London - Hampstead Research Ethics Committee.

23. Further information and contact details

You are encouraged to ask any questions you wish, before, during or after your treatment. If you have any questions about the study, please speak to your study nurse or doctor, who will be able to provide you with up to date information about the drug involved. If you wish to read the research on which this study is based, please ask your study nurse or doctor. If you require any further information or have any concerns while taking part in the study please contact one of the following people:

Your Study Doctor

Name*:* ***[insert name]*** Tel. Number: ***[insert number]***

For peer review only - http://bmjopen.bmj.com/site/about/guidelines.xhtml

Your Research Nurse

Name; ***[insert name]*** Tel. Number: ***[insert number]***

Emergency Contact Number: [*insert number*]

If you decide you would like to take part then please read and sign the consent form. You will be given a copy of this information sheet and the consent form to keep. A copy of the consent form will be filed in your patient notes and one will be filed with the study records. You can have more time to think this over if you are at all unsure. Thank you for taking the time to read this information sheet and to consider this study.

For peer review only

For peer review only - http://bmjopen.bmj.com/site/about/guidelines.xhtml

[to be printed on hospital headed paper]

Study title: BEAT LUPUS- Safety and efficacy of BElimumab After B cell depleTion therapy in systemic LUPUS erythematosus

Protocol Reference Number: CTU/2013/096

Participant Identification Number: ___________

CONSENT FORM

Please initial each line

1	I have read the information sheet version ______ dated ________________for this study. I have had the opportunity to consider the information and ask questions that have been answered satisfactorily.	____________
2	I understand that my participation in this study is voluntary and that I am free to withdraw at any time, without giving any reason and without my medical care or legal rights being affected.	____________
3	I agree to my General Practitioner being informed of my participation in this study.	____________
4	I understand that relevant sections of my medical notes and data collected during the study may be looked at by individuals from the sponsor of the study (University College London), regulatory authorities, or from the NHS Trust, where it is relevant to my taking part in this research.	____________
5	I agree to take part in the above study.	____________
6	I agree that specimens collected as part of the above study may be transferred outside of the hospital in which they were taken, for storage and analysis for this and other ethically approved studies in the future.	____________
7	I agree to provide contact details for a family member, carer or friend to be used in case the study team are unable to contact me for follow up appointments.	____________

Name of Participant	Date	Signature
Name of Witness (where necessary)	Date	Signature
Name of Researcher	Date	Signature

When completed: 1 for participant; 1 for researcher site file (original); 1 to be kept in medical notes

For peer review only - http://bmjopen.bmj.com/site/about/guidelines.xhtml

SPIRIT

STANDARD PROTOCOL ITEMS: RECOMMENDATIONS FOR INTERVENTIONAL TRIALS

SPIRIT 2013 Checklist: Recommended items to address in a clinical trial protocol and related documents*

**Section/item**	**Item No**	**Description**	**Page Number on which item is reported**
**Administrative information**			
Title	1	Descriptive title identifying the study design, population, interventions, and, if applicable, trial acronym	1
Trial registration	2a	Trial identifier and registry name. If not yet registered, name of intended registry	2
	2b	All items from the World Health Organization Trial Registration Data Set	Throughout the manuscript
Protocol version	3	Date and version identifier	14
Funding	4	Sources and types of financial, material, and other support	18
Roles and responsibilitie s	5a	Names, affiliations, and roles of protocol contributors	1 and 18
	5b	Name and contact information for the trial sponsor	18
	5c	Role of study sponsor and funders, if any, in study design; collection, management, analysis, and interpretation of data; writing of the report; and the decision to submit the report for publication, including whether they will have ultimate authority over any of these activities	18
	5d	Composition, roles, and responsibilities of the coordinating centre, steering committee, endpoint adjudication committee, data management team, and other individuals or groups overseeing the trial, if applicable (see Item 21a for data monitoring committee)	14
**Introduction**			

For peer review only

Background and rationale	6a	Description of research question and justification for undertaking the trial, including summary of relevant studies (published and unpublished) examining benefits and harms for each intervention	4
	6b	Explanation for choice of comparators	4-5
Objectives	7	Specific objectives or hypotheses	2
Trial design	8	Description of trial design including type of trial (eg, parallel group, crossover, factorial, single group), allocation ratio, and framework (eg, superiority, equivalence, noninferiority, exploratory)	6-14
**Methods: Participants, interventions, and outcomes**			
Study setting	9	Description of study settings (eg, community clinic, academic hospital) and list of countries where data will be collected. Reference to where list of study sites can be obtained	1, 10
Eligibility criteria	10	Inclusion and exclusion criteria for participants. If applicable, eligibility criteria for study centres and individuals who will perform the interventions (eg, surgeons, psychotherapists)	7
Interventions	11a	Interventions for each group with sufficient detail to allow replication, including how and when they will be administered	6
	11b	Criteria for discontinuing or modifying allocated interventions for a given trial participant (eg, drug dose change in response to harms, participant request, or improving/worsening disease)	8
	11c	Strategies to improve adherence to intervention protocols, and any procedures for monitoring adherence (eg, drug tablet return, laboratory tests)	Not applicable
	11d	Relevant concomitant care and interventions that are permitted or prohibited during the trial	9
Outcomes	12	Primary, secondary, and other outcomes, including the specific measurement variable (eg, systolic blood pressure), analysis metric (eg, change from baseline, final value, time to event), method of aggregation (eg, median, proportion), and time point for each outcome. Explanation of the clinical relevance of chosen efficacy and harm outcomes is strongly recommended	11

For peer review only - http://bmjopen.bmj.com/site/about/guidelines.xhtml

Participant timeline	13	Time schedule of enrolment, interventions (including any run-ins and washouts), assessments, and visits for participants. A schematic diagram is highly recommended (see Figure)	6-8
Sample size	14	Estimated number of participants needed to achieve study objectives and how it was determined, including clinical and statistical assumptions supporting any sample size calculations	11-13
Recruitment	15	Strategies for achieving adequate participant enrolment to reach target sample size	Not applicable
**Methods: Assignment of interventions (for controlled trials)**			
Allocation:			
Sequence generation	16a	Method of generating the allocation sequence (eg, computer-generated random numbers), and list of any factors for stratification. To reduce predictability of a random sequence, details of any planned restriction (eg, blocking) should be provided in a separate document that is unavailable to those who enrol participants or assign interventions	8
Allocation concealment mechanism	16b	Mechanism of implementing the allocation sequence (eg, central telephone; sequentially numbered, opaque, sealed envelopes), describing any steps to conceal the sequence until interventions are assigned	8
Implementation	16c	Who will generate the allocation sequence, who will enrol participants, and who will assign participants to interventions	8
Blinding (masking)	17a	Who will be blinded after assignment to interventions (eg, trial participants, care providers, outcome assessors, data analysts), and how	8
	17b	If blinded, circumstances under which unblinding is permissible, and procedure for revealing a participant's allocated intervention during the trial	8
**Methods: Data collection, management, and analysis**			

Data collection methods	18a	Plans for assessment and collection of outcome, baseline, and other trial data, including any related processes to promote data quality (eg, duplicate measurements, training of assessors) and a description of study instruments (eg, questionnaires, laboratory tests) along with their reliability and validity, if known. Reference to where data collection forms can be found, if not in the protocol	10 and Box 2
	18b	Plans to promote participant retention and complete follow-up, including list of any outcome data to be collected for participants who discontinue or deviate from intervention protocols	Not applicable
Data management	19	Plans for data entry, coding, security, and storage, including any related processes to promote data quality (eg, double data entry; range checks for data values). Reference to where details of data management procedures can be found, if not in the protocol	10
Statistical methods	20a	Statistical methods for analysing primary and secondary outcomes. Reference to where other details of the statistical analysis plan can be found, if not in the protocol	12-13
	20b	Methods for any additional analyses (eg, subgroup and adjusted analyses)	12-13
	20c	Definition of analysis population relating to protocol non-adherence (eg, as randomised analysis), and any statistical methods to handle missing data (eg, multiple imputation)	12-13
**Methods: Monitoring**			
Data monitoring	21a	Composition of data monitoring committee (DMC); summary of its role and reporting structure; statement of whether it is independent from the sponsor and competing interests; and reference to where further details about its charter can be found, if not in the protocol. Alternatively, an explanation of why a DMC is not needed	14
	21b	Description of any interim analyses and stopping guidelines, including who will have access to these interim results and make the final decision to terminate the trial	14

For peer review only - http://bmjopen.bmj.com/site/about/guidelines.xhtml

Harms	22	Plans for collecting, assessing, reporting, and managing solicited and spontaneously reported adverse events and other unintended effects of trial interventions or trial conduct	13-14
Auditing	23	Frequency and procedures for auditing trial conduct, if any, and whether the process will be independent from investigators and the sponsor	10
**Ethics and dissemination**			
Research ethics approval	24	Plans for seeking research ethics committee/institutional review board (REC/IRB) approval	13
Protocol amendments	25	Plans for communicating important protocol modifications (eg, changes to eligibility criteria, outcomes, analyses) to relevant parties (eg, investigators, REC/IRBs, trial participants, trial registries, journals, regulators)	13
Consent or assent	26a	Who will obtain informed consent or assent from potential trial participants or authorised surrogates, and how (see Item 32)	13
	26b	Additional consent provisions for collection and use of participant data and biological specimens in ancillary studies, if applicable	Not applicable
Confidentiality	27	How personal information about potential and enrolled participants will be collected, shared, and maintained in order to protect confidentiality before, during, and after the trial	10
Declaration of interests	28	Financial and other competing interests for principal investigators for the overall trial and each study site	18
Access to data	29	Statement of who will have access to the final trial dataset, and disclosure of contractual agreements that limit such access for investigators	14
Ancillary and post-trial care	30	Provisions, if any, for ancillary and post-trial care, and for compensation to those who suffer harm from trial participation	Not applicable
Dissemination policy	31a	Plans for investigators and sponsor to communicate trial results to participants, healthcare professionals, the public, and other relevant groups (eg, via publication, reporting in results databases, or other data sharing arrangements), including any publication restrictions	10

For peer review only

	31b	Authorship eligibility guidelines and any intended use of professional writers	14
	31c	Plans, if any, for granting public access to the full protocol, participant-level dataset, and statistical code	Not applicable
**Appendices**			
Informed consent materials	32	Model consent form and other related documentation given to participants and authorised surrogates	Enclosed as a supplementary file
Biological specimens	33	Plans for collection, laboratory evaluation, and storage of biological specimens for genetic or molecular analysis in the current trial and for future use in ancillary studies, if applicable	Not applicable

*It is strongly recommended that this checklist be read in conjunction with the SPIRIT 2013 Explanation & Elaboration for important clarification on the items. Amendments to the protocol should be tracked and dated. The SPIRIT checklist is copyrighted by the SPIRIT Group under the Creative Commons "Attribution-NonCommercial-NoDerivs 3.0 Unported" license.

For peer review only
